# Genome-wide mutagenesis of *Zea mays *L. using *RescueMu *transposons

**DOI:** 10.1186/gb-2004-5-10-r82

**Published:** 2004-09-23

**Authors:** John Fernandes, Qunfeng Dong, Bret Schneider, Darren J Morrow, Guo-Ling Nan, Volker Brendel, Virginia Walbot

**Affiliations:** 1 Department of Biological Sciences, Stanford University, Stanford, CA 94305, USA; 2 Department of Genetics, Development and Cell Biology, Iowa State University, Ames, IA 50011, USA; 3 Department of Statistics, Iowa State University, Ames, IA 50011, USA

## Abstract

Derived from the maize *Mu1 *transposon, *RescueMu *provides strategies for maize gene discovery and mutant phenotypic analysis. 9.92 Mb of gene-enriched sequences next to *RescueMu *insertion sites were co-assembled with expressed sequence tags and analyzed. Multiple plasmid recoveries identified probable germinal insertions and screening of *RescueMu *plasmid libraries identified plants containing probable germinal insertions. Although frequently recovered parental insertions and insertion hotspots reduce the efficiency of gene discovery per plasmid, *RescueMu *targets a large variety of genes and produces knockout mutants.

## Abstract 2

The authors describe a large-scale transposon-tagging study in maize using the *RescueMu* system. The study provides a large resource of tagged and sequenced maize alleles, as well as some insights into the biology of *RescueMu*.

## Background

*MuDR/Mu *transposable elements are widely used for mutagenesis and as tags for gene cloning in maize [1,2]. The high efficiency of *Mu *insertional mutagenesis regulated by *MuDR *in highly active Mutator lines reflects four features of this transposon family. First, a plant typically has 10-50 copies of the mobile *Mu *elements [3], although some plants have over 100 copies. Second, they insert late in the maize life cycle, generating diverse mutant alleles transmitted in the gametes of an individual Mutator plant [1]. Third, they exhibit a high preference for insertion into genes [1]. And fourth, most maize genes are targets as judged by the facile recovery of *Mu *insertion alleles in targeted screens [1,4-6]. In directed tagging experiments, the frequency of *Mu*-induced mutations for a chosen target gene is 10^-3^-10^-5 ^[7]. Interestingly, a *bronze1 *exon [8] and the 5' untranslated region of *glossy8 *[9] contain hotspots for *Mu *insertion in specific regions, which may explain the higher frequency of mutable allele recovery for these genes.

Somatic mutability, visualized as revertant sectors on a mutant background, is indicative of transposon mobility. By monitoring maintenance of a mutable phenotype, it was established that the Mutator transposon system is subject to abrupt epigenetic silencing, which affects some individuals in most families [10,11]. A molecular hallmark of silencing is that both the non-autonomous *Mu *elements and the regulatory *MuDR *element become hypermethylated [12,13]. Without selection for somatic instability of a visible reporter allele and/or hypo-methylation, Mutator lines inevitably lose *Mu *element mobility.

The high efficiency of *Mu *mutagenesis has been exploited in several reverse genetics strategies. The first protocol described used PCR to screen plant DNA samples to find *Mu *insertions into specific genes using one primer reading out from the conserved *Mu *terminal inverted repeats (TIRs) and a gene-specific primer [14-17]. Alternatively, survey sequencing of maize genomic DNA flanking *Mu *insertions yields a list of tagged genes in each plant [18,19]. A third method uses *RescueMu*, *a Mu1 *element containing a pBluescript plasmid, to conduct plasmid rescue by transformation of *Escherichia coli *with total maize DNA samples. To identify insertions in genes of interest, *RescueMu *plasmids can be screened or the contiguous host genomic DNA can be sequenced using primers permitting selective sequencing from the right or left TIRs of *Mu1 *[20].

Here we describe the initial results of a large scale *RescueMu *tagging effort conducted by the Maize Gene Discovery Project. The tagging strategy employed grids of up to 2,304 plants organized into 48 rows and 48 columns. Plasmid rescue was undertaken from individual pools of up to 48 plants per row or column. Genomic sequences next to *RescueMu *insertion sites were obtained for all the rows and for a subset of columns of six grids. Maize genomic sequences were subsequently assembled into 14,887 unique genomic loci using computational approaches. These loci were analyzed for gene content, the presence of repetitive DNA and correspondence to mapped maize genes and ESTs. Gene models were built by co-assembling the genomic sequence with ESTs and cDNAs by spliced alignment and by *ab initio *gene prediction. Identified gene models were tentatively classified using gene ontology terms of potential homologs [21].

Many features of *Mu *element behavior have been examined previously using hundreds of tagged alleles or by analyzing the population of *Mu *elements in particular plants and a few descendants. With single founder individuals for the analyzed tagging grids, we could examine the distribution of new insertion sites of *RescueMu *in large progeny sets. The contiguous genomic sequences were analyzed to determine if there were insertion hotspots, preferential insertion site motifs, routine generation of the expected 9-base-pair (bp) direct target sequence duplication (TSD) and evidence of pre-meiotic insertion events.

Like other *Mu *elements, *RescueMu *exhibits a strong bias for insertion into or near genes, as few insertions were recovered in retrotransposons or other repetitive DNA. In addition, for the set of *RescueMu *insertions into confirmed genes, a bias for insertions into exons (rather than introns) was observed, consistent with the well-established use of Mutator as a mutagen. The gene-enrichment exhibited by *RescueMu *was compared against two physical methods of gene enrichment, methyl filtration [22] and high *C_0_t *genome fractionation [23].

## Results

### *RescueMu *transposition in active Mutator lines

**Figure 1:**
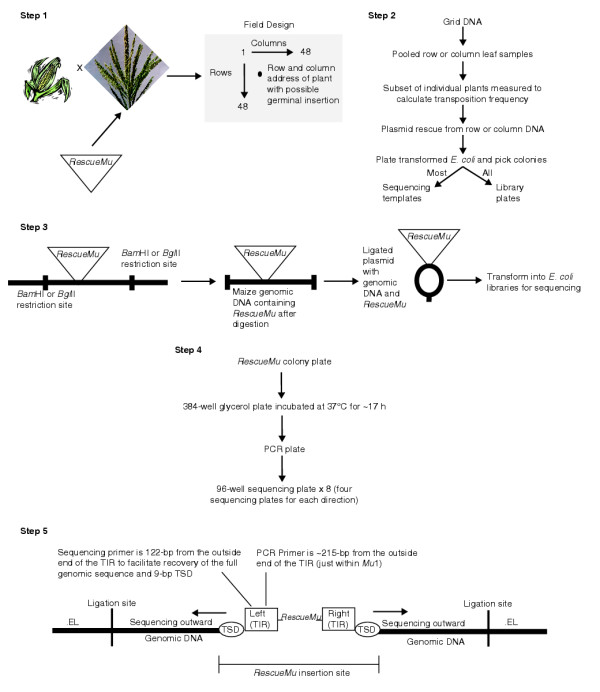
Schematic diagram of *RescueMu *grid tagging and sequencing (*RescueMu *not to scale). Step 1: *RescueMu *is introduced into embryogenic callus followed by crossing of regenerated plants to active Mutator lines. Lines are screened for transposed *RescueMu *elements in plants lacking the original transgene array. Pollen from one *RescueMu *donor plant is crossed to multiple ears of a non-*RescueMu *line to generate tagging grids of up to 48 rows × 48 columns of *trRescueMu *plants in the field. Step 2: plant DNA prepared from pools of row or column leaves is used to generates transformed bacterial libraries of *RescueMu *plasmids. These are used as sequencing templates and for construction of a library plate representing the diverse insertion sites in grid plants. Step 3: genomic DNA is digested using two restriction enzymes (*Bam*HI, *Bgl*II), religated into plasmids and transformed into *E. coli*. Step 4: after transformation, *RescueMu *plasmid-containing *E. coli *colonies are selected by plating onto carbenicillin agar plates and picked into 384-well plates with growth/freezing media. Overnight incubation is followed by a PCR reaction designed to amplify longer inserts with lengths up to 16 kb. Using the PCR product, eight 96-well sequencing plates (four for sequence from the left TSD and four from the right TSD) are created. Step 5: priming strategy and relative locations of PCR and sequencing primers within the *RescueMu *element. The sequencing reactions are read out from the TSDs to recover the germinal insert sequence. Although a *Bam*HI and *Bgl*II double-restriction digest produces a shorter, easier-to-sequence insert length, it also increases the ambiguity in interpreting the sequence during analysis. Given successful sequencing in both directions, two GSS sequences may be submitted for every plasmid (sequence flanking the left and right TIRs). Two additional GSS sequences may be submitted for a plasmid when a *Bam*HI, *Bgl*II or *Bam*HI-*Bgl*II ligation site is encountered. Each of these occurrences yields sequence that was not necessarily contiguous *in vivo*. Dubious GSS sequences are designated with the suffix .1EL (re-created enzyme ligation site) or .2EL (re-created enzyme ligation of two restriction sites not encountered *in vivo*). Sequence flanking TIRs *in vivo *is submitted as GSS sequences with no suffix except the .x or .y (right or left) direction designation.

In standard Mutator lines, *Mu1 *elements maintain copy number through successive outcrosses, indicating that some type of duplicative transposition occurs [24] in the absence of genetic reversion [25]. Most new mutations are independent and occur late in the life cycle [26,27]. Consequently, a single pollen donor can be used to generate thousands of progeny with diverse *Mu *insertion events (Figure 1). Initially *RescueMu *germinal insertions were sought by direct mobilization of elements from transgene arrays containing multiple copies of the original *35S:RescueMu:Lc *plasmid and the plasmid conferring resistance to the herbicide Basta used for selection of transformed callus [20]. Using eight different transgene arrays crossed with diverse active Mutator lines, the average germinal transposition frequency through pollen was only 0.07 (Table 1, grid A); lines with a single *MuDR *element had no transposed *RescueMu *(*trRescueMu*).

Materials were selected from the progeny of grid A plants for grids B through E using two criteria: there were visible seedling mutations in around 10% of progeny characteristic of a very active Mutator line [26] and the presence of *trRescueMu*. By DNA blot hybridization of individuals within grids B through E, the *RescueMu *transposition frequencies ranged from 0.1 to 0.26 (Table 1). By sequence analysis after plasmid rescue, *trRescueMu *were identified that had inserted into likely maize genes and generated the diagnostic 9-bp TSD characteristic of *Mu *transposition (data not shown). There were also events initially scored as transposition by blot hybridization that represented *RescueMu *rearrangements within the transgene array, and deleted forms of *RescueMu *were detected by blot hybridization and gel electrophoretic sizing of rescued plasmids (data not shown). Although *RescueMu *insertion frequency was low, overall *Mu *movement was very high in these grids; visible, independent seedling mutations were identified in 10.1-28.3% of the selfed progeny (Table 1), as high as the most active Mutator lines described to date [28].

**Figure 2:**
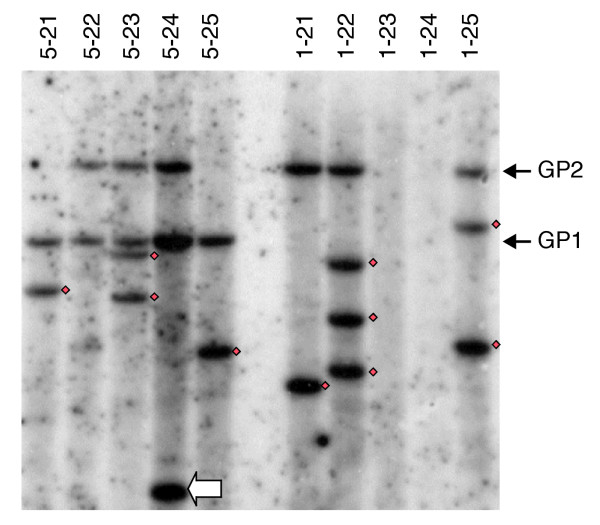
DNA blot hybridization analysis of *trRescueMu *elements in grid G. Total DNA was prepared from individual grid G plants in rows 1 and 5, as listed at the top of the lanes; these rows represent two ears crossed by the same founder *RescueMu *pollen source. DNA samples were digested with *Hin*dIII, a unique site 0.5 kb from the internal end of the left TIR of the *RescueMu *element, and the resulting gel blot was hybridized with an ampicillin-resistance gene fragment to visualize *RescueMu*. The two parental *trRescueMu *had been identified in the founder plant, and these size classes are marked along the right side of the autoradiogram. Hybridizing bands corresponding to new *trRescueMu *are indicated with a black square; the hybridizing band too small to be a full-length *trRescueMu *is marked with a white arrow. GP, grid G parental insertion sites 1 and 2 shown to be segregating in the progeny.

In an effort to increase transposition frequency, lines with *trRescueMu *but no transgene array were selected. Plants with a verified *trRescueMu *were crossed to *r-g *and colorless kernels selected - these lack red spotting from *RescueMu *somatic excision from the *35S:RescueMu:Lc *transgene. During subsequent plant growth Basta-sensitivity was scored as a second indicator that the transgene array was absent [20] and DNA blot hybridization then confirmed that a *trRescueMu *but not the Basta-resistance transgene was present in the plant. To guard against Mutator silencing, plants were also screened by DNA blot hybridization to verify that they contained unmethylated *Mu1 *and *MuDR *elements after digestion of genomic DNA with the methylation-sensitive enzymes *Hin*fI and *Sst*I, respectively (data not shown). Four plants each with a single *trRescueMu *were identified by these criteria and crossed to *r-g*. A DNA blot hybridization screen was conducted on 393 progeny of these four individuals. Seven progeny were identified with two new *trRescueMu*, seven plants were identified with three events, and 33 plants had a single *trRescueMu*; the original, parental *trRescueMu *elements were shown to segregate as Mendelian factors in the populations screened (data not shown). The 14 plants with two or three new *trRescueMu *were each crossed by an anthocyanin tester and also crossed multiple times as pollen parents to tester lines to generate sufficient progeny to construct one grid from each founder plant. Inexplicably, in sampling seedling progeny from each outcross ear, some lineages had very few new *trRescueMu*. The lines with the highest transposition frequencies had two *trRescueMu *and were used in grids G through J; DNA blot hybridization analysis of 30-200 grid plants was used to estimate transposition frequencies within each grid, which ranged from 0.38 to 0.66 (Table 1), with an average of 0.58 per plant and 0.29 per parental *RescueMu *element. The two parental *trRescueMu *elements were shown to be segregating 1:1 and independently (Figure 2 for grid G, and data not shown for other families).

Subsequently, surveys within each grid were used to identify plants with two or three newly *trRescueMu *and no evidence of Mutator silencing for construction of the next tagging populations. In this manner, the frequency of *trRescueMu *was increased in some grids to 1.0-1.4 per plant (Table 1) reflecting a frequency of 0.5-0.7 per parental element.

### Library plate preparation and gene representation

As shown schematically in Figure 1, the *trRescueMu *insertion sites have been immortalized by preparing libraries from each of the row and column leaf pools from 16 grids, with three additional grid libraries under construction (Table 1). Briefly, total maize DNA was digested with *Bam*HI and *Bgl*II, both of which recognize sites outside of *RescueMu*, and the fragment mixture was used to transform *E. coli *(see Materials and methods). The resulting library plates contain 56-96 individual row and column libraries representing the diversity of germinal *trRescueMu *and a sampling of somatic events present in the harvested leaf tissue (each well in a library plate is a pool of 20-48 plants from a row or column). The parental *RescueMu *insertion sites inherited from the grid founder(s) are present in every library.

Library plates contain a high diversity of genomic sequences. In a row of 48 plants, assuming random insertion, two segregating founder elements and a transposition frequency of 1.0, there will be 50 different plasmid types in the heritable class. Including heritable and somatic insertions, we estimate that each row or column library contains about 100-200 distinct plasmid types. Given these parameters, a library plate from a 48 row × 48 column grid with an average of 150 somatic plasmids per row or column library would contain 14,400 somatic insertion sites plus 2,304 germinal events and the two parental insertion sites. Because *RescueMu *shows a strong bias for insertion into genes [20], each library plate contains a substantial fraction of the predicted 50,000 genes of maize [29], provided the insertion sites are random. Ultimately, library plates for 19 grids derived from 33,000 plants and containing an estimated 30,108 heritable *trRescueMu *insertion sites (grid size × transposition frequency from Table 1) will be available online from the Maize Gene Discovery project through MaizeGDB [30].

### Plasmid recovery analysis and identification of probable germinal insertions (PGIs)

Based on gel electrophoretic analysis of nearly 1,000 rescued plasmids, the genomic DNA flanking *RescueMu *averaged 3.5 kilobases (kb), with a range of 0.4-15 kb (data not shown). To accommodate the large size of some plasmids, a PCR template preparation protocol was devised to amplify genomic inserts of up to 16 kb for high-throughput sequencing [31]; primers were designed to amplify from within the right and left TIRs reading outward into the maize genomic DNA such that high quality sequence would be available to identify the TSDs flanking *RescueMu *insertion sites. Plasmids from all rows plus several columns of a grid were sequenced, with a routine yield of 80-92% success. A subset of plasmids could not be bidirectionally sequenced, because they lacked the TIRs at one or both ends. Deleted forms of *trRescueMu *were detected in several percent of the individuals surveyed by DNA blot hybridization (see Figure 2 for an example). If such derivatives retained the origin of replication and ampicillin-resistance marker, they could be cloned by plasmid rescue; if the TIRs were absent, they could not be sequenced.

Previous analysis of *trRescueMu *demonstrated that somatic insertion events, typically found in a tiny leaf sector, were sequenced just once from a leaf DNA sample while multiple instances of the germinal events could be recovered [20]. Out of 28,988 non-parental plasmids sequenced, 41% (11,749) were recovered once (new *trRescueMu *somatic plus germinal insertion events) for each grid, and 59% (17,239) were recovered multiple times (probable new *trRescueMu *germinal insertion events). In addition, a total of 24,875 parental plasmids were transmitted from the founder plants. The percentage of parental plasmids within each grid varied from 17% for grid G to 61% for grid P. Some grids had more parentals than other grids and some parental plasmids were preferentially sequenced for unknown reasons. The parental insertion sites include the two or three known parental sites that each segregated into 50% of the progeny. Somatic sectors in the tassel or ear of the parental plant that generated plasmids found in multiple individuals within the grid are analyzed in a later section.

Grid sequence data were used to cross-check the transposition frequency estimated from DNA blot hybridization (Table 1) using both a row and column matching method and a more general multiple recovery method. Analysis of 80 individuals from six contributing outcross ears in grid G identified 54 that were newly *trRescueMu*, equivalent to a frequency of 0.68 new insertions per plant. Using a Poisson model based on this transposition frequency for an individual grid (Table 1), the sequencing goal was established to reach a depth sufficient to insure that with 95% confidence, each probable germinal insertion would be recovered at least once. In the Poisson model, the 5% probability for the zero class (in other words, the 95% probability of finding all PGIs at least once) occurs when the observed mean is -ln(0.05) or approximately 3. After sequencing several rows and at least one column for a grid, multiple occurrences of PGIs were counted and used to project the sequences required to obtain the desired average of 3 occurrences of each PGI. As a cross-check of this coverage using the row and column matching method, the sequenced row plasmids were compared to the sequences available from four columns of grid G and 149 matches were found. This is equivalent to a transposition frequency of 0.81 based on 149/(4 × 46 plants per row), somewhat higher than the estimate of 0.68 based on blot hybridization analysis of individual plants. Recovery in both a row and a column is highly indicative of a probable germinal insertion because the row and column plasmids were obtained from different leaves and only germinal insertions would be found throughout a plant. The results for each analyzed grid are shown in Table 2. The low column sampling in grid K (only 192 plasmids were attempted for each of three columns) and grid M (96 plasmids for two columns and 192 plasmids for a third column) resulted in a lower than expected number of germinal insertions. Grid P had a low germinal insertion count with this method because a portion of the column sequences was from rows generated from different parental plants and subsequently excluded from the analysis.

Analysis of the row and column sequence data within grids demonstrates that the row sequencing was too shallow to recover some probable germinal insertions more than once and that a fraction of germinal insertions were not sequenced. For example, within grid G, 385 plasmids were identified twice in the available column data but were missing from the row sequences; this is over twice the number of plasmids identified by row and column matching. From the number of plasmids successfully sequenced per row within grid G, we estimated a 70-95% probability of sequencing the likely germinal insertion events at least once in the rows. For other grids, the sampling efficiency ranged from 30 to 95% per row. Grids in which some rows had sampling efficiency less than 60% are listed as partial in Table 1; sequencing was terminated in portions of these grids because of technical difficulties such as an excess representation of a parental insertion site, a large number of rearranged *RescueMu *elements that could not be sequenced with the standard protocol, or poor yield of *RescueMu *plasmids for unknown reasons.

The second method of identifying probable germinal insertions includes plasmids that were recovered multiple times, regardless of whether a column sequence was present. Almost all somatic insertions should only be recovered once due to their occurrence in just a few cells. The results using this method for each grid are shown in Table 3.

**Figure 3:**
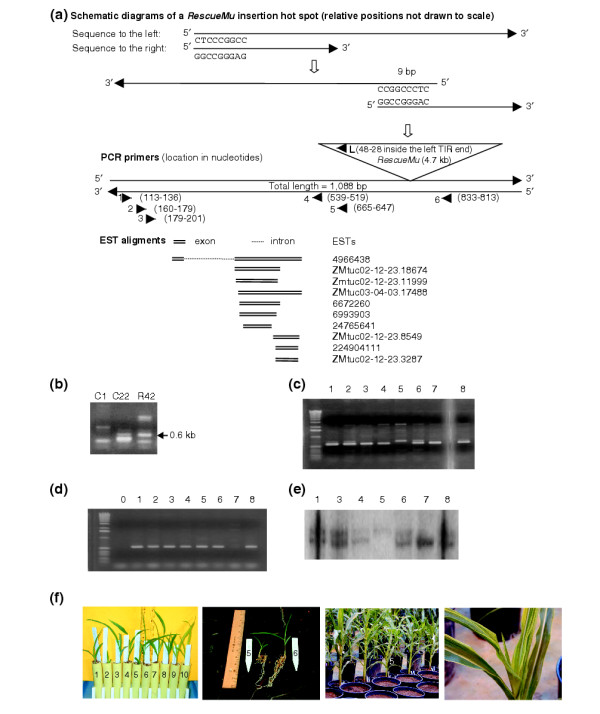
*RescueMu *plasmid library plate screening for a gene with multiple insertion sites. **(a) **Schematic diagrams of a *RescueMu *insertion hotspot: demonstration of the assembly of flanking genomic sequences; locations and directions of all primers used in this study; EST alignment to genomic sequence assembly showing introns. **(b) **An ethidium bromide-stained agarose gel of the PCR products from columns 1 and 22 and row 42 plasmid libraries, using primer pair 2 + L. **(c) **An ethidium bromide-stained agarose gel of the PCR products with leaf DNA extracted from G42-22(x) progeny 1 to 8, using primer pair 3 + 6. **(d) **An ethidium bromide-stained agarose gel of the PCR products with the same DNA used in **(c)**, except using primer pair 3 + L (column B is blank). **(e) ***Nco*I-digested DNA blot from plants 1 and 3 to 8 probed with a fragment spanning a 0.6-kb PCR product amplified with primer pair 1 + 5. **(f) **Phenotypes at several developmental stages (from left to right): 10-day-old seedlings (1 to 10 from left to right) of the G42-22(x) progeny; a side-by-side comparison of plants 5 and 6 at 10 days, including their root mass; adult plants at 1 month showing plant 5 in the foreground of the picture with two siblings on either side; a close-up of the plant 5 adult leaf phenotype.

What these data mean in practice is that the 3,138 probable germinal insertions identified after sequencing the same *RescueMu *plasmid at least twice is not a comprehensive list of the heritable insertion events. On the basis of the number of grid plants and estimated transposition frequencies (Table 1), 8,311 probable germinal insertions were expected from the six grids (see Table 3). From this we estimate that the majority of the heritable insertion events are represented by only a single sequenced *RescueMu *plasmid. It is likely that nearly half of the plasmids recovered just once represent a germinal insertion (0.44 = (8,311-3,138/11,749)). By PCR screening of library plates containing the immortalized row and column plasmids, plants containing a specific insertion event can be verified (Figures 1 and 3). Selection against specific plasmids in *E. coli *probably contributed to non-recovery of certain insertion sites as sequencing templates, and these plasmids may also be under-represented in library plates.

### Verification of germinal transmission

Individual grid plants with probable germinal insertions were identified on the basis of recovery of the same plasmid in both a row and a column. In addition, library plates containing all of the row and column libraries can be screened using PCR, with one primer designed to the *Mu1 *TIRs present in *RescueMu *and a second primer in the gene of interest, as illustrated in Figure 3. A probable germinal insertion plasmid should yield the same size product in at least one row and one column library of that grid plate; the row and column identifiers specify the address of the plant(s) containing this insertion. To test this method, 11 instances of duplicate plasmid recovery in grid G (N. Arnoult and G-L.N., unpublished data) and 14 such cases in grid H (K. Goellner and V.W., unpublished data) were verified to be represented in both a row and a column library by PCR screening of the corresponding library plate. Seedling progeny from the identified row and column plants were evaluated for the presence of the expected *RescueMu *insertion site. A germinal insertion was verified for 16/16 cases examined by DNA blot hybridization and/or PCR of individual progeny plants in the family (see Additional data file 2 for methods and for plants used to verify germinal transmission [31]).

### Mutational spectrum of *RescueMu*

**Figure 4:**
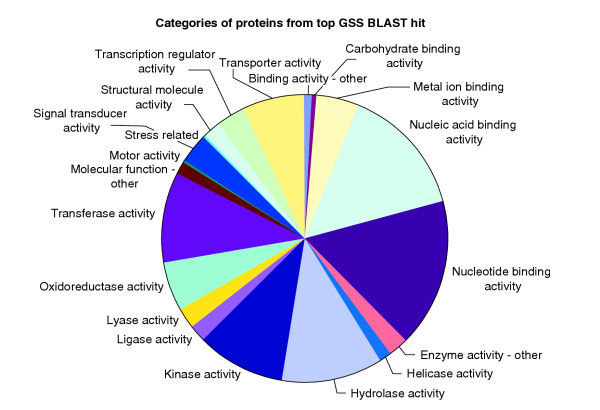
Functional spectrum of genes targeted by *trRescueMu*. Functional spectrum of probable proteins, identified by BLASTX of GSS contigs against the SPTR database, for trRescueMu targeted genes. Functional categories were derived from the Gene Ontology (GO) database.

As shown in Figure 4, *RescueMu *insertions occur in diverse gene types. Illustrating the utility of *Mu *tagging, insertions are found in housekeeping genes, such as actin, as well as in regulatory genes such as those for transcription factors and protein kinases. Using the database of mapped maize genes and expressed sequence tags (ESTs) [30], *RescueMu *insertions are identified in genes on all 10 maize chromosomes [32]. These data confirm earlier studies tracking *Mu *insertions using DNA blot hybridization that established that these elements insert throughout the genome and do not show a measurable bias for insertion locally [1]. In addition, about 85% of *RescueMu *insertion sites that match maize ESTs correspond to genes of unknown function, suggesting the discovery of novel genes.

Of the 14,887 *RescueMu *insertion sites identified in six grids (multiple insertions into a gene from the same grid being counted only once because the majority are the same insertion event), 88% represent single instances of transposon insertion locations. There were 596 instances of a specific genomic sequence having two or more *RescueMu *insertion events. If the maize genome contains 50,000 distinct genes that are targets of *Mu *insertional mutagenesis, then far fewer cases of duplicate recovery would be expected by chance alone, given the number of events analyzed (*p *< 0.001); therefore, *RescueMu *exhibits some preference for particular genes.

To determine if there were 'hotspots' for *RescueMu *insertion within particular genes, data were compared between grids with independent founder individuals. As summarized in Table 4, 90% of the *RescueMu *insertion sites were found in just one grid. This was true for both probable germinal insertion events (plasmids found two or more times within a grid) as well as for singlet sites (a mixture of germinal and somatic events). The 10% of insertion sites found in two or more grids represent independent recovery of a *RescueMu *insertion into the same locus.

In addition to the computational comparison in which an overlap of 50 bases (95% identity) was scored as insertion into the same gene, over 730 insertion sites were examined manually for 250 cases of genes with insertions from more than one grid. Of these insertion sites, 80% were at different locations within the same locus; we found 85 cases of insertions within a 1-10 bp region and 67 cases of insertions at the same base. Previously, Dietrich *et al*. [9] reported that 62 of 75 *Mu *insertions at *glossy8 *were in the 5' untranslated region, with 15 insertions at the same base; similarly, the beginning of exon 2 within *bronze1 *is the most frequent site of *Mu *insertion in that gene [8].

One *RescueMu *contig from the Genomic Survey Sequencing (GSS) section of GenBank, ZM_RM_GSStuc03-10-31.4765 [33], is a hotspot for *RescueMu *insertion, with six plasmids sequenced from row 42 of grid G and one each from grids H, I, and M. Insertion sites were identical across the grids. Sequences generated to both the left and right of the *RescueMu *element were aligned as demonstrated in Figure 3a. Many maize ESTs matching a maize acetohydroxyacid synthase were found near this insertion site; the closest (GenBank GI: 4966438) is less than 50 bp away. Because this *RescueMu *insertion site was recovered multiple times in grid G, a heritable insertion may exist. After PCR screening of grid G plasmid libraries, summarized in Figure 3a, the plant at row 42, column 22 was identified. To assess heritability of this *RescueMu *insertion site, total leaf DNA was extracted from selfed seed of this plant, namely G 42-22, obtained from the Maize Genetics Cooperation Stock Center. PCR screening of the DNA (Figure 3c) indicated that plant 5 is homozygous for the insertion and plant 7 is homozygous wild type. DNA blot hybridization with a 0.6-kb purified PCR probe amplified with primer pair 1 + 5 confirmed plant 5 to contain the homozygous insertion allele, plant 7 to be wild-type, and the rest to be heterozygous for the insertion (Figure 3e). Various mutant phenotypes were observed in plant 5 (Figure 3f), including retarded seedling growth, reduced plant height, discolored streaks on adult leaves and sterile tassel and ear. Because there are multiple *Mu *elements in this line, further characterization of selfed progeny of its heterozygous siblings will be performed to determine the true phenotype caused by this insertion.

### Analysis of 9-bp TSD and insertion site preferences

Because a 9-bp TSD is characteristic of *Mu *insertion events, the 9 bp next to the left and right TIRs of an individual *RescueMu *plasmid were used to join the right and left flanking sequence provided they were complementary (Figures 1, 3); note that the sequences are complementary because they were generated from different strands. For non-parental plasmids, left and right sequence data were available for 13,966 plasmids, and the 9 bp was readily identified computationally for 47.2% (6,596) of these. The remaining non-parental plasmids did not have both right and left sequence data and/or the 9-bp motif could not be verified; 5.7% (1,816) contain only post-ligation sequences. Possible explanations for incomplete sequencing results include deletions next to *Mu1 *elements that remove a portion of the TIR as well as flanking host sequence [34,35]; these events occur with about a 10^-2 ^frequency at existing insertion sites and if they occurred during or subsequent to *RescueMu *insertion they would preclude identification of the 9-bp repeat. Alternatively, the lack of a 9-bp TSD could reflect sequencing error. Manual inspection of 300 of the unmatched cases indicated that for nearly 90% there was an 8/9-base repeat match with the mismatch being an undetermined base (an 'N') or a single missing or additional base. Given that all sequences were single pass but of high average quality (phred 35, equivalent to one base-calling error in 3,160 bases), we consider that 9-bp TSDs exist in virtually all *trRescueMu *insertion sites. A few cases showed anomalies in the TSDs, which probably reflect rearrangements near *RescueMu*.

Several groups have reported weak consensus insertion site preferences for *Mu *based on smaller data sets [9,18,20]. We have derived a site-specific frequency profile of the bases from 3,999 *RescueMu *insertion regions [32]. The profile is in agreement with what has been reported earlier by Dietrich *et al*. [9], showing a strong bias for high G/C content in the 9-bp TSD within a flanking dyad-symmetrical consensus: CCT-(TSD)-AGG. The non-random insertion pattern strongly suggests that *RescueMu *targeting is at least partially dependent on sequence features. In addition, we have compared the profiles derived independently from insertion sites within confirmed exons, introns and uncharacterized regions, respectively, and found the same base preferences in all three sets (data not shown).

Of 14,887 genomic loci, 62% matched maize or other plant EST/cDNAs. As more genomic sequence becomes available that can be assembled with ESTs to annotate the non-coding portions of maize genes, it will be interesting to determine if the *RescueMu *insertion sites that do not match an EST or gene in another species represent introns or other non-coding genic regions. On the basis of the gene structure annotated by maize EST matching, we have located 968 TSD sites within genes. Of these, 849 are inside exons. To check if *RescueMu *has a preference for insertion into exons (that is, the above observed high frequencies of exon insertions is not the result of potential high exon proportion in the maize genes), a standard binomial test with normal approximation was performed. On the basis of the matching to ESTs, the lengths of all exons and introns observed from all *RescueMu *contigs were counted as 2,182,954 bp and 439,403 bp, respectively. Assuming that *RescueMu *does not have a preference to insert into exons (null hypothesis), the probability of observing an exon insertion event is proportional to the length of exons (single binomial trial probability 0.832). The probability of observing at least 849 exon insertion events was calculated (less than 0.001; reject the null hypothesis). This result suggests that *RescueMu *has some preference to target exon regions within genes.

As outlined in Materials and methods, the *RescueMu *GSS sequences were scanned and masked for repetitive elements as collected in The Institute for Genomic Research (TIGR) Cereal Repeat Database [36]. The repeat content was compared with results for GSS sequences derived by methylation filtration (MF) and high *C_0_t *selection (HC) using the same repeat-masking criteria [36]. The percentage of masked nucleotides was 16.5, 24.5 and 16.2% for *RescueMu*, MF and HC, respectively.

Therefore, on the nucleotide level, *RescueMu *shows similar repeat content as the physical enrichment methods. However, after we assembled the *RescueMu *GSS sequences to remove redundancy, only about 3% of the *RescueMu *loci are composed of repetitive DNA (equal or greater than 75% masked, Table 5). If the maize genome is two-thirds retroelements [37], then there is an approximately eightfold insertion bias by *RescueMu *against this component of the genome. We also downloaded the latest MF and HC contigs (version 3.0) from TIGR [38] and applied the same repeat masking on those contigs. Our results show that 28% of the MF and 6% of HC contigs are repetitive DNA. Thus, *RescueMu *and HC have similar bias against repetitive DNA, superior to the MF bias. It should be noted, however, that the MF and HC GSS sequencing has generated, on average, much longer contigs than *RescueMu *(see Additional data file 2).

In addition, only 0.4% of the *RescueMu *insertions were found in either the approximately 10,000 copies of the 9.1 kb 28S + 18S rRNA genes [39] comprising 3.6% of the 2.5 gigabase (Gb) maize genome, or in the large number of tRNA and 5S rRNA genes in the maize genome (Table 5). These results demonstrate a strong bias against insertion into genes transcribed by RNA polymerases I and III.

Also shown in Table 5, about 62% of the *RescueMu *loci match strongly to maize or other plant ESTs or appear to encode proteins with high similarity to known proteins. In addition, about another 5% of the loci were predicted to be genic regions with high stringency by *ab initio *gene prediction programs. As a control, we matched ESTs to contigs assembled from unfiltered (random) maize GSS sequences [38]. From about 33,000 of those unfiltered contigs, less than 20% of them show significant matching to ESTs. This shows that *RescueMu *contigs contain more than threefold enrichment of genic regions than random sequencing. This is consistent with our expectation that *RescueMu *preferentially inserts into genes. It is worth pointing out that plant EST collections contain ESTs from repetitive elements. Although we masked contigs using the annotated TIGR repeat database [38], it is possible that some contigs still contain unidentified repetitive elements, which might overestimate the number of genic regions by matching the same ESTs to different copies of repetitive elements. In particular, 18% of the EST matched regions show high similarity to transposon coding regions based on BLAST searches against the GenBank nucleotide and protein databases, suggesting that at most 14% of unfiltered contigs include protein-coding genes. The numbers of genic sequences from MF and HC was reported to be 27% and 22%, respectively [36]. However, these numbers are not directly comparable to our *RescueMu *results, because these authors used much higher stringency for the EST spliced alignments with the BLAT program [40], requiring 95 and 80% identity, respectively, when matching to the TIGR maize gene index or other plant indices. We used the GeneSeqer program for spliced alignment of the *RescueMu *data, which tolerates less sequence matching without compromising gene structure prediction accuracy [41]. The results using GeneSeqer for *RescueMu*, MF, and HC are very similar (data not shown).

Palmer *et al*. [42] evaluated the gene discovery rates of MF, EST sequencing and *RescueMu *by comparing the respective sequence sets to rice gene models. They concluded that unique gene discovery is most efficient with MF at a sequencing depth when EST sampling saturates. However, their reported low gene discovery rate for *RescueMu *does not reflect the *RescueMu *insertion bias, because their dataset included all sequences deposited in GenBank. That is, they did not remove the redundancy resulting from multiple sequencing of parental insertions.

### Multiply recovered *RescueMu *insertion sites in the progeny of a single founder plant

Probable germinal insertions involve plasmids recovered several times within a sequenced row and/or column, but in addition, some *RescueMu *insertion sites were found in two or more row libraries (Table 6). Although these could represent hotspots for *Mu *insertion at exactly the same base, we consider it more likely that they reflect the known ability of *Mu *elements to insert pre-meiotically, resulting in several progeny with the same newly generated mutation present as a sector on an ear indicative of a single insertion event [43,44]. Robertson estimated that 20% of *Mu *transpositions occur pre-meiotically, 60% occur during meiosis or immediately afterwards, and 20% occur after the mitosis that separates the two sperm in haploid pollen [1]. We infer that multiple row recovery of the same insertion site within a grid was indicative of a likely pre-meiotic insertion; in contrast, authentic hotspots have the same insertion site among grids. A second line of evidence is that DNA blot hybridization surveys to calculate transposition frequency within a grid identified many instances of a particular fragment size shared in two or more progeny (data not shown). Finally, phenotypic screening of grid progeny families identified numerous instances of identical phenotypes segregating in related families [45]; each such phenotypic class was counted just once in calculating the percentage of families with a new visible phenotypic mutation (Table 1).

To calculate the extent and timing of pre-meiotic sectors, the sequenced plasmids from grids G, H, I, K, M and P were classified as occurring in a single row or in multiple rows. The development of the tassel and ear must be considered when evaluating these data. An insertion event that occurs during meiosis can be represented in two haploid cells. During microgametophyte (haploid plant) ontogeny, both of these cells survive, resulting in two pollen grains with the same event. In contrast, only one megagametophyte develops after megaspore meiosis; therefore, female meiotic and subsequent events in the haploid megagametophyte are always represented in just one progeny plant. Most grid plants resulted from male transmission of *RescueMu *and a minority (about 10%) from female transmission. Given that the founder plants produced copious pollen, there is a low probability that two grains carrying the same meiotic insertion will both result in seed; therefore, the same *RescueMu *insertion site found in two rows should usually be from a pre-meiotic transposition event. For all events found in three or more rows, the insertion event must be pre-meiotic.

The 103 insertions sites found in three or more rows of grid G must be pre-meiotic events (see Table 6). They represent 9% of the probable germinal insertion events (103/1,091) identified by the criterion of recovery of the same plasmid twice or more (see Table 3). The percentage was similar for all six grids: there were 321 events identified in three or more rows out of 3,138 probable germinal insertions. Surprisingly, 138 contigs were found in four or more rows in these six grids, including 34 events in 10 or more rows (Table 6). Therefore, occasionally there is a *RescueMu *insertion event very early in the somatic development of the inflorescence or in the apical meristems. The majority of *trRescueMu *insertion sites are found in only one row (92% of germinal plus somatic insertion sites, Table 6).

As a cross-check on the analysis of pre-meiotic events presented in Table 6, we evaluated the actual number of individual plants containing the same insertion site for a subset of each grid, using the sequence data from columns. Using this method we confirmed that among 184 plants in grid G with both row and column sequence data, there were 65 cases of insertion sites found in two or more rows or in two or more columns (Table 7). Similar results were obtained for the other five grids. From these calculations and the data in Table 6 it appears that *RescueMu *insertions must occur routinely before meiosis and that, although rare, there are a significant number of early somatic insertion events that are transmitted to multiple progeny.

## Discussion

*RescueMu *was introduced into maize by particle bombardment resulting in complex transgene loci containing multiple copies of the transposon and the Basta-resistance plasmid used for selection of transgenic lines [20]. After crossing with an active Mutator line, *RescueMu *exhibited somatic excision from a *35S:Lc *reporter allele resulting in a red-spotted aleurone but the heritable insertion frequency was very low. Progeny screening identified individuals containing two or three *trRescueMu *elements lacking the original transgene array by genetic segregation and unmethylated *Mu1 *and *MuDR *elements. Some of these individuals and subsequent derivatives with the same characteristics were used as founder plants to construct grids of plants organized into rows and columns for efficient generation and analysis of germinal mutations. Tagging maize sequences with *RescueMu *followed by plasmid rescue and sequencing of the flanking host DNA has identified 3,138 insertion locales from 17,239 plasmids (see Table 3). These plasmids represent 59.5% (17,239/28,988) of the total non-parental plasmids of the genomic loci found in each grid. Because sequencing depth was too shallow to identify all likely germinal insertions, the 40.5% of non-parental plasmids recovered just once (11,749 from Table 3) represent a mixture of somatic and germinal events. On the basis of the estimation of germinal insertion frequency from DNA blot hybridization, the six grids should contain more than 8,000 heritable *trRescueMu *insertion sites, but the sequencing depth was too shallow to identify all of these by multiple recovery of the same plasmid two or more times.

*RescueMu *is suited for both reverse and forward genetic strategies. Given the genomic sequence contiguous to any *trRescueMu*, a PCR screen can be designed to identify which plant contains the insertion of interest using 96-well plates containing the immortalized collection of row and column rescued plasmids. The row and column plant address can be used to order seed for further genetic and phenotypic analysis as illustrated by the *RescueMu *insertion into the acetolactate synthase gene (Figure 3). Alternatively, the phenotype database, which is organized by individual plant, can be searched to identify individuals segregating for mutations of interest. Active Mutator lines with multiple mobile *Mu *elements were used so most mutations will be caused by these *Mu *elements because they increase mutation frequency 50-100-fold above spontaneous levels [1]. The high forward mutation frequency reflects the copy number of the elements and their preference for insertion into or near transcription units [1]. From the DNA hybridization blots (data not shown) used to verify that grid founder plants had unmethylated *Mu *elements, the copy number of unmethylated *Mu *elements was estimated at 20-40 per founder; therefore, two mobile *RescueMu *elements would be expected to account for 5-10% of the newly generated mutations. Seed was ordered through the Maize Genetics Cooperation Stock Center [46] for further characterization.

*RescueMu *insertions were found in genes and ESTs mapped to all 10 maize chromosomes [31], and were found in all of the gene classifications for maize (Figure 4). These data confirm the empirical observations of maize geneticists that *MuDR/Mu *transposons are general and efficient mutagens for maize genes [1]. Analysis of 14,887 loci defined by *RescueMu *insertions demonstrates that transposition is highly preferential for RNA polymerase II transcription units: about 62% of the sites match maize or plant ESTs. Because the EST collections are incomplete and lack intron and promoter sequences, it is likely that an even higher proportion of *RescueMu *insertion sites are in or near genes but cannot be currently assigned to a specific gene. Given the current efficiency, large tagging populations in excess of 200,000 plants would be required in order to recover *RescueMu *mutations in all maize genes (estimation is based on the calculation method in [47]). The numerous grids evaluated for phenotypic characteristics should approach saturation of visible mutations, although most of the mutations are caused by standard *Mu *elements.

Given that the maize genome comprises approximately 70% retrotransposons and other highly repetitive sequences, including around 10,000 copies of the rRNA genes [37], these components of the maize genome are significantly under-represented in *RescueMu *insertion sites. Only about 8% of the *RescueMu *insertion sites match repetitive elements and few insertions (0.4%) were recovered in genes transcribed by RNA polymerase I or III. These results suggest that a chromatin component associated with polymerase II transcription units or the absence of a structure in other classes of genes is important in targeting *RescueMu *and other *Mu *elements to maize genes. Similarly, recombination during meiosis and transcription *per se *is targeted to genes. It is likely that the parasitic *Mu *elements exploit an element of host gene packaging that evolved for other reasons to facilitate transposition into genes.

The biological specificity for maize genes exhibited by *RescueMu *is close to methyl filtration and high *C_0_t *fractionation. The probable germinal insertion class defines a collection of mutations of enormous potential for the phenotypic characterization of maize with specifically disrupted functions. However, the low cost of template production is a distinct advantage of both physical enrichment methods compared to the high cost of designing, sampling and self-pollinating tagging grids. Current levels of sample sequencing from the physical enrichment templates highlight the desired redundancy of the *RescueMu *method, which is important for distinguishing somatic from germinal insertions at individual loci. The physical enrichment methods are considerably below one times coverage of the transcriptome of around 250 Mb; hence the current efficiency of generating novel sequence (the likelihood that the next clone sequenced is new) is much higher with these methods than with *RescueMu*.

Using the *RescueMu *insertion site data, several parameters of *Mu *transposition behavior were investigated. We confirm that a 9-bp TSD is characteristic of virtually all *Mu *insertion sites. We confirm that a small percentage of *trRescueMu *suffer deletions, including loss of a TIR, as noted in previous studies of *Mu1 *[35]. Through evaluation of several hundred *Mu *insertion sites [9,18], consensus motifs have been proposed for insertion sites. The sequence profile derived from the much larger population of *RescueMu *insertion sites is consistent with the previously proposed motifs. A bias exists for G+C-rich sequence, reflecting the composition of maize exons. We confirm that there are hotspots for *Mu *insertion, identified by finding identical *trRescueMu *insertion sites in independent grids. A few loci were recovered in four or more of the six grids analyzed, and many more in two (1,295 genes) or three (233 genes) grids. There is no strong DNA consensus motif at these hotspots, and we consider it more likely that a specific DNA structure or a protein associated with genes establishes conditions for efficient *Mu *insertion at particular sites. It is important to note that active transcription is not a requirement for *Mu *element insertion; otherwise *Mu *would preferentially insert into genes active late in floral development and in gametophytes.

The *trRescueMu *insertion sites represent a mixture of non-heritable somatic insertions present in leaves, germinal insertions in single grid individuals, insertion events in pre-germinal sectors within flowers, and parental elements. Parental elements identified in a grid founder plant segregated 1:1 in the progeny as expected. In addition, some insertion events were found in three or more grid rows, and hence in three or more individuals, and must be pre-meiotic transposition events in the founder. This class represented 10.2% (321/3,138) of all the likely germinal insertions identified (calculated from Table 6). Given the clonal analysis model of the pattern of cell divisions establishing the ear and tassel of maize [48-50], the earliest events within the apical meristem could affect up to half of the ear or tassel, with subsequent events affecting progressively narrower portions of the inflorescence. The majority of the pre-meiotic events are consistent with *RescueMu *transposition in the floral cells a few cell divisions before the onset of meiosis, that is, in precursor cells that are still proliferating and could generate at least two and up to approximately 50 meiocytes. A smaller fraction of new insertions events occurred early enough to be represented in many progeny of a particular plant. These rare, early transposition events generate very large sectors within the developing inflorescence.

*Mu *transposon mutagenesis is highly efficient, primarily because the transposon targets genes and it is usually found in 10-50 copies per genome. How does the plant tolerate the large number of mutations generated by this agent? Within the diploid somatic tissues, most new mutations lack a phenotype; however, the haploid gametophytes are subject to stringent selection. Unlike animals, in which the phenotypes of the sperm and egg are set by previous gene activity in the parent, many characteristics of the haploid phase of the plant life cycle reflect haploid genetic activity, which requires overlapping but distinctive suites of genes in the mega- and microgametophytes [51]. Consequently, the late timing of new *Mu *insertions generates gamete diversity, but the unfit genotypes are culled from the population before fertilization. Coe *et al*. [52] describe the general problem that lethals occur much more frequently in pollen than in the megagametophyte. Any method that relies on pollen transmission will therefore fail to recover certain types of mutations that would be recovered through female transmission. For this reason, a subset of maize genes required in both types of gametophyte is refractory to knockout mutagenesis.

## Conclusions

A public resource of transposon-tagged maize alleles was constructed and evaluated. *RescueMu *is an efficient tag for mutagenizing and cloning maize genes, because 66% of insertion sites appear to be in genes. Sequencing from immortalized plasmid libraries organized into row and column plates reflecting the organization of fields of plants permit identification of probable germinal insertions; the library plates can be searched by PCR to verify germinal insertions and subsequently acquire seed of the corresponding plant. Alternatively, a searchable database of segregating plant phenotypes in seed, seedling, or adult tissues can be used to find plants carrying mutations of interest. Although *RescueMu *can target most, if not all, RNA polymerase II transcription units in the nuclear genome, the transposon does exhibit hotspots in particular genes. Neither the hotspots nor other insertion sites contain a motif(s) defining predictable insertion locations. *RescueMu *properties confirm attributes established with smaller populations of standard *Mu *elements.

## Materials and methods

### Biological materials

*RescueMu *contains all of *Mu1 *plus a 400-bp segment of *Sinorhizobium meliloti *and pBluescript (Stratagene), as described previously by Raizada *et al*. [20]. The complete sequence of *RescueMu *was obtained in this study using PCR primers to amplify overlapping sections of the element [31] for bidirectional sequencing (GenBank accession AY301066). In the construct used to make transgenic plants, the *RescueMu *transposon was placed in the 5' untranslated region of a *35S:Lc *expression plasmid where it blocked expression [20]. *Lc *is a member of the R family of transcriptional regulators of the anthocyanin pathway [53]. Transgenic maize lines in the A188 × B73 (*r-r/r-g*, *A1*, *Bz1*, *Bz2*) hybrid background were crossed to *r-g *testers and subsequently with *r-g *Mutator lines containing multiple copies of *MuDR *to visualize *RescueMu *somatic excision as red anthocyanin sectors in an otherwise white aleurone. The tagging populations used here were developed by screening for transposition of *RescueMu *from the original, complex transgene arrays to diverse genomic locations. Using DNA blot hybridization, these once-transposed *RescueMu *(*trRescueMu*) were closely monitored for subsequent transposition, and lines were monitored for *Mu1 *and/or *MuDR *methylation in the TIRs, a sign of incipient Mutator silencing. Details of line development and evaluation, including DNA blot hybridization methods, will be presented elsewhere. The anthocyanin tester lines (recessive for *r-g*, *a1*, *bz1 *or *bz2*) were in inbreds W23, K55, A188, or hybrid combinations of these lines. Some *RescueMu *lines used in tagging grids were crossed to inbreds A619 or B73, which are both *r-g*, *A1*, *Bz1*, *Bz2*. Grid backgrounds are presented in detail at [31].

### Plasmid rescue and DNA sequencing

Detailed protocols are presented at [54], and a schematic is provided in Figure 1. Briefly, leaf tissue was collected from all plants in each row and from a different leaf in each column of a grid. A separate plasmid rescue library was constructed after *Bam*HI plus *Bgl*II digestion of the genomic DNA preparations. These libraries were immortalized in library plates available from the project [31]. Plated colonies were picked, grown overnight in liquid media, and sequencing templates prepared by a direct PCR method suitable for amplifying genomic inserts of up to 16 kb. Cycle sequencing was performed using Big Dye Terminator chemistry to read out from a position around 110 within the left or right terminal inverted repeat (TIR) of *RescueMu*; although the primers were selective for one TIR, there was some cross-priming. All grid rows plus several columns were sequenced. Three 96-well plates were normally sequenced for each row or column to obtain sequence information for a desired minimum of 200 plasmids; additional sequencing reactions were conducted if necessary. Matches of row and column sequences are designated as probable germinal insertions, because they represent an insertion site present in two leaves of that plant (designated by its row and column address); when only row sequences were available from a particular plasmid, probable germinal insertions were designated after recovery of the same sequence two or more times. Plasmid types recovered just once are a mixture of heritable and strictly somatic insertions. Parental *RescueMu *insertion sites present in a grid founder plant segregated in the grid progeny, and these insertion sites were expected to be found in all rows and columns. In some cases, particular parental plasmids were over-represented in the sequenced plasmid population. To reduce their contribution and increase recovery of new insertion sites, a rare-cutting restriction enzyme site was identified in the parental plasmid and the corresponding enzyme was included in the genomic DNA preparation to bias against recovery of that parental plasmid.

PCR screening of a library plate to quantify a *RescueMu *insertion hotspot Six gene primers plus one *RescueMu *left readout primer were used in this study:

1. 5'-TTGGGAGGTTGAAGGTAAAGACAT-3'

2. 5'-GTGCTG GATTGGTTACTCCG-3'

3. 5'-CGATGATTCTAGTTGAGCGTCTG-3'

4. 5'-ACTCGCACCAACATGAATACC-3'

5. 5'-GTTTCCGAGGACGCAGAGG-3'

6. 5'-AGCGCCAGGGCCAGGGGATTC-3'

L. 5'-CAT TTC GTC GAA TCC CCT TCC-3' (*RescueMu*)

Locations and directions with respect to the insertion site of *RescueMu *are shown in Figure 3a. PCR conditions were as follows: 5-20 ng of each plasmid library, 2.0-2.5 mM Mg^2+^, 0.4 mM dNTPs, 0.8-1.0 μM gene primer and 4-5 μM *RescueMu *L primer in a 50 μl reaction was first denatured for 2 min at 95°C followed by 35 cycles of 30 sec at 95°C, 30 sec at 55°C and 2 min at 72°C, and a final 2 min extension at 72°C. The same PCR conditions were used for screening using 5-100 ng samples of maize total genomic DNA.

### DNA blot hybridization

Total genomic DNA was extracted from leaf tissues using a modified urea method [55]. After overnight digestion, the restricted DNA was separated on a 0.8% agarose gel and transferred onto Hybond-N+ membrane (Amersham Biosciences) in 0.4 M NaOH. Blots were hybridized with non-radioactive probes labeled with AlkPhos DIRECT system (Amersham Biosciences) for chemiluminescence detection on X-ray film.

### Initial clustering and assembly of genomic sequences

The sequences were screened to remove the TIR sequences using the program crossmatch [56] and then trimmed to achieve a minimum phred score >15 in sliding windows over 40 bases. Overall the quality scores averaged phred >35, or less than one error in 3,160 bases. The average length of the trimmed, high quality genomic sequence entering the assembly was 378 bases. The right-TIR primer yielded 22% more successful sequence than the left-TIR primer resulting in an excess of right side sequences. Trimmed sequences were then assembled into contigs using phrap [56] with the following parameters: -minmatch 35 -minscore 30 -node_seq 14 -node_space 9. The member sequences for each contig were extracted from the phrap output files and assigned to a row or column of a grid. Within each contig, only a single sequence from a plasmid was used to determine the row and column representation. For example, if both the left- and right-flanking sequence from a plasmid assembled into one contig, this was considered one recovery of the plasmid. If the left-flanking sequence from one plasmid and the right-flanking sequence from a separate plasmid assembled into the same contig, this was considered two independent recoveries of the same genomic locus. In the latter case, if the right- flanking sequence was from a different row, then the sequence was recovered in multiple rows as well. All sequences were deposited into the Genomic Survey Sequencing (GSS) section of GenBank [57].

### Assembly of *RescueMu*-derived genomic sequence data

As shown in Figure 1, using the 9-bp TSD characteristically generated during *Mu *element insertion [1], the sequences to the right and left of a particular *RescueMu *element can be assembled into a continuous sequence. To do this, trimmed *RescueMu *GSS sequences were downloaded from GenBank [58], for comparison to raw GSS sequences containing the *Mu1 *TIR sequences. The TIRs were masked by the cross_match program [56] to determine the flanking 9-bp TSD sequences. The TSDs are the end-overlaps between GSS sequences generated from the left and right side of *RescueMu *insertion. Merging through TSDs using the reverse-complementary strand of the left and right sequences recovers the original genomic sequences flanking the *RescueMu *insertion. A special consideration in the assembly of the genomic sequences flanking the right- and left-TIRs of *RescueMu *is the presence of a GGATCC (*Bam*HI), AGATCT (*Bgl*II), or a GGATCT (*Bgl*II/*Bam*HI) or AGATCC (*Bam*HI/*Bgl*II) motif. The two restriction digestion sites represent a true ligation site of sequence that was non-contiguous in the maize genome, but the post-restriction site sequences can unambiguously be assigned to the right or the left of *RescueMu*. On the other hand, the GGATCT or AGATCC motif could be contiguous genomic sequence or could have been generated during the ligation step of the plasmid rescue. Consequently, assignment of the position of the sequence beyond the GGATCT or AGATCC motif is ambiguous. If the *RescueMu *insertion site matched EST sequence across and beyond the GGATCT or AGATCC motif, the post-ligation sequence could be properly assigned (Figure 1). In the *RescueMu *plasmid sequences considered here, the average number of sequences reported to GenBank was 2.3 (131,364/57,022) per plasmid.

The 131,364 *RescueMu *GSS sequences deposited at GenBank were screened for vector sequences against the UniVec database at the National Center for Biotechnology Information (NCBI) [59] using the crossmatch program: -mismatch 12 -penalty -2 -minscore 20. The resulting 130,861 vector-trimmed sequences were then screened against the maize repeat database annotated by TIGR [60] using the Vmatch program [61] with the parameters -l 50 -h 3 -identity 95. The 127,708 repeat-free sequences were then used to identify parental insertions. Any given *RescueMu*-transformed plant contains the parental *RescueMu *elements that were recovered at a high frequency during sequencing (from every sequenced row or column). Because our goal is to analyze the gene discovery by newly inserted *RescueMu *(that is, we are interested in where those non-parentals inserted into the maize genome), we decided to filter out the parental sequences as much as possible. We used Vmatch to cluster near-identical left and right sequences for each grid. A parental cluster contains sequences from nearly all the row or column sequences. A total of 59,069 parental sequences were identified and were excluded from the subsequent assembly. All the non-parental sequences were first preassembled for each plasmid using the left and right 9-bp TSD overlap. The merged GSSs were first clustered by PaCE [62] (minimum exact match 36 bp, minimum score threshold 30%) and then consensus sequences (contigs) for each cluster were generated by CAP3 [63] (overlap 40 bp; 90% identity cutoff). Because PaCE and CAP3 only pair sequence with the minimal overlap required to establish statistically significant identity, the number of contigs is probably an overestimate of the number of independent *RescueMu *insertion sites. For the particular case where TSDs were not recovered during sequencing, the left and right sequences could not be assembled together, even though they were from the same plasmid. Therefore, a Perl script was developed to conduct single-linkage clustering based on clone-pair constraints to assemble the GSS to the same 'genomic loci' if they were derived from the same plasmid clone.

### Classification of insertion site context

To be successful as a gene-discovery tool, the transposon insertions must be predominantly into the genic regions of the maize genome. To quantify the potential enrichment of the *RescueMu *flanking sequences for genic regions, we matched all assembled contig sequences against various classes of known repetitive sequences, including retrotransposons, DNA transposons, centromeric and telomeric repeats, rRNA genes and plastid DNA. For this analysis, the non-parental sequences were used in their original form, with only vector sequences but not repeat sequences trimmed. The sequences previously discarded for analysis because they consist almost entirely of repetitive elements were assembled using the same procedure as described above for the repeat-trimmed sequences. Note, however, that this number of loci is unreliable and probably an underestimate of the true number of loci recovered because of the intrinsic difficulty with assembling repetitive DNA. To identify the repetitive elements in the contigs, Vmatch (-seedlength 14 -hxdrop3 -l 30 -identity 70) was used in combination with the TIGR cereal repeat database (version 2 consisting of maize, rice, barley, sorghum and wheat repeats). The contigs were also scanned from tRNA genes by tRANscan-SE program [64] with its default parameters.

### Gene discovery in GSS contigs

Both similarity-based and *ab initio *approaches have been used to detect gene structures of the GSS contigs. For the similarity-based approach, GeneSeqer [65] programs were used to match plant EST contigs and cDNAs to GSS contigs. The plant EST contigs were regularly assembled by PlantGDB [66]. For the *ab initio *prediction, GENSCAN [67] (with default parameter settings for maize) was used and only high exon score predications (≥0.90) were selected. The GSS contigs were compared against SPTR [68], a nonredundant protein data set collected by the European Bioinformatics Institute (EBI), using BLASTX [69] with an E-value ≤ e-20. The BLASTX top protein hits were used to assign putative functions to the unique regions and for classification into functional categories based on annotation in the Gene Ontology [21] database.

The genetically mapped maize ESTs were retrieved from MaizeGDB [70]. These ESTs were spliced-aligned to GSS contigs using GeneSeqer as described above. The matched GSS contigs were then plotted on the maize IBM Neighbor genetic map [30].

### Analysis of 9-bp TSD and insertion site preferences

For the analysis of *RescueMu *target sites, we retrieved the 9-bp TSD sequences from the confirmed insertion sites where both the left and right sequences match on the 9-bp TSD. We also retrieved the 20 bp up- and downstream sequences around the TSD. Then a 15-base long profile (9-base TSD and its three up- and downstream neighbors) was derived from the sequences and their reverse-complement orientation determined using the Expectation Maximization Algorithm [71].

### Analysis of tentative unique contigs containing GSS sequences from multiple grids

The GSS seqeunces present in each tentative unique contig (TUCs) were extracted from [31] and assigned to a row or column within a grid. A sample of TUCs with GSS sequences from multiple grids was then selected for detailed analysis. For each GSS in the TUC (excluding post-ligation sequences), the exact location of the TSD was determined by visual examination of the sequence alignment file for the TUC and the untrimmed GSS sequence data. The number of GSS sequences for each grid at each transposition site was recorded.

### Phenotypic analysis

Grid plants were self-pollinated unless male or female-sterile. The resulting F1 families were evaluated by inspection of ears and kernels, at weekly intervals for five weeks after germination in a sand bench in a greenhouse, and at weekly intervals throughout the life cycle in the field. Phenotypes observed were recorded and are assembled into a searchable database at [31]. Unique phenotypes were documented with a digital image, and there are links to corresponding *RescueMu *flanking sequences where established. Instructions on how to obtain seed of grid plants is also provided.

## Additional data files

The following additional data are available with the online version of this article: a table listing the internal primers used in sequencing *RescueMu *(Additional data file 1), supplementary material for this paper, including details of methods (Additional data file 2).

## Supplementary Material

Additional data file 1 A table listing the internal primers used in sequencing *RescueMu* Click here for additional data file

Additional data file 2 Supplementary material for this paper, including details of methods Click here for additional data file

## Figures and Tables

**Table 1:** Grid organization and analysis of mutant phenotypes segregating among selfed progeny of grid plants

Grid*	Year^†^	Grid size^‡ ^(row × col)	Plasmid rescued	Libraries sequenced^§^	Transposition frequency^¶^	Independent mutations (% of families)^¥^	
						
						Seed	Seedling
A	1999H	34 × 48	No	No	0.07	7.2	4.5
B	1999SD	52 × 48	No	No	0.10	8.6	10.1
C	1999B	40 × 40	No	No	0.13	8.3	28.3
D	1999S	48 × 48	No	No	0.26	8.7	15.1
E	2000H	40 × 48	No	No	0.25	8.6	27.0
F	2000AZ	41 × 41	No	No	0.57	6.6	19.5
G	2000S	46 × 48	Yes	Yes	0.68	5.0	11.9
H	2000B	38 × 36	Yes	Yes	0.62	7.5	6.9
I	2000B	38 × 34	Yes	Yes	0.62	9.5	9.8
J	2000SD	38 × 45	Yes	Survey	0.38	9.8	11.1
K	2001H	30 × 30	Yes	Yes	0.66	8.0	20.3
L	2001H	36 × 20	Yes	Yes	0.66	12.8	17.4
M	2001AZ	40 × 40	Yes	Partial	1.30	8.2	ND
N	2001B	32 × 44	In progress	No	0.20	6.3	ND
O	2001S	47 × 48	Yes	Survey	0.50	5.2	ND
P	2002H	48 × 48	Yes	Yes	1.40	5.9	ND
Q	2002H	48 × 24	Yes	Yes	1.00	2.7	ND
R	2002AZ	36 × 36	Yes	Survey	0.72	3.7	ND
S	2002SD	48 × 48	Yes	Survey	1.00	12.7	ND
T	2002H	48 × 46	Yes	Survey	1.00	ND	ND
U	2002H	48 × 48	Yes	Partial	>1.30	ND	ND
AA	2002S	48 × 48	Yes	Yes	0.60	ND	ND
BB	2001B	34 × 48	Yes	Survey	0.60	6.2	ND
V	2003AZ	45 × 45	In progress	Survey	1.00	ND	ND
X	2003SD	44 × 44	In progress	Survey	1.00	ND	ND

*Grids with a single letter contain mainly plants with a *RescueMu *pollen parent plus the seed from the ear of the founder male crossed by a non-Mutator line. In grids with a double letter, both parents contained *RescueMu*. ^†^Summer nurseries are designated by year and location: A, Tucson, AZ; B, Berkeley, CA; SD, San Diego, CA; S, Stanford, CA. H indicates the winter Hawaii nursery. ^‡^Vandalism, animals, and environmental damage in the field resulted in some losses compared to expectation of the ear harvest. Ears with fewer than 100 kernels and those from outcross pollinations of male or female sterile grid plants were not assessed for mutation frequency; these lines are being propagated at the Maize Coop by sib pollination to establish a permanent line for later evaluation and distribution. ^§^Yes, indicates that all rows plus four columns were sequenced with the goal of coverage to a depth such that there was a 80-95% probability that plasmids representing germinal insertions would be identified at least once. Grids listed as partial have limited (40%-80%) depth from some rows. Survey sequencing was performed on several rows and columns on the indicated grids to verify that plasmids organized into library plates contained authentic *trRescueMu*. Library plates will be available from all grids, including V and X, during 2004 as listed at [31]. ^¶^Frequency of newly transposed *RescueMu *per plant based on DNA blot hybridization, sampling 30-200 plants per grid. For grid A only, the data are from plants sibling to those in the grid. ^¥^Progeny families generated by self-pollination of grid plants were examined for kernel defects before shelling, and seedling traits were scored on up to 30 surviving individuals grown from each family. A minimum of 200 families were scored for the seedling forward-mutation frequency, and all selfed ears were scored for the seed defects. Mutations were scored as independent if they were not segregating in multiple families from the same founder. Phenotypic descriptions are available at [31], and it is expected that the grids not yet analyzed (ND) and the summer 2003 grids V, W, and X will be scored during 2004 and 2005 for reporting through the project database. Most mutations are caused by standard *Mu *elements.

**Table 2:** Probable germinal insertions (PGI) based on row and column matches

Grid	Rows (r)	Columns (c)	Transposition frequency (τ)*	Expected PGI (e)^†^	Row + column matches (m)	Percentage of expected^‡^	Transposition frequency (using row + column)^§^
G	46	4	0.68	125.1	149	119%	0.81
H^¶^	36	4	0.62	89.3	115	129%	0.80
I	38	5	0.62	117.8	128	109%	0.67
K	30	3	0.66	59.4	32	54%	0.36
M^¶^	40	3	1.30	156.0	33	21%	0.28
P	37	4	1.40	207.2	71	34%	0.48
Total				754.8	528	70%	

*Expected frequency of PGI was determined from DNA gel blot analysis of frequency of newly transposed *RescueMu *per plant as stated in Table 1; ^†^expected = r × c × τ; ^‡^percentage of expected = 100 × m/e; ^§^transposition frequency = m/(r × c); ^¶^for grids H and M, rows were considered columns and vice versa to simplify calculations.

**Table 3:** Probable germinal insertions (PGI) based on multiply recovered plasmids

Grid	Multiple recovery (m)	Single recovery (s)	Percentage PGI*	Expected frequency (τ)^†^	Expected PGI (e)^‡^	Percentage of expected^§^	Plasmids in multiple recoveries
G	1,091	3,801	22%	0.68	1,501	73%	5,535
H	535	2,142	20%	0.62	848	63%	2,945
I	544	2,000	21%	0.62	801	68%	3,162
K	228	1,000	19%	0.66	594	38%	1,202
M	330	1,075	23%	1.3	2,080	16%	2,053
P^¶^	410	1,731	19%	1.4	2,486^¶^	16%	2,342
Total	3,138	11,749	21%		8,311	38%	17,239

Single recoveries are also shown. *Percentage of PGI = m/(m + s); ^†^expected frequency of PGI was taken from Table 1; ^‡^expected PGI = τ × rows × columns (see Table 2); ^§^percentage of expected = 100 × m/e; ^¶^based on 37 rows only.

**Table 4:** Detailed analysis of insertion sites recovered multiple times

Number of same-base insertions that occurred in the indicated number of grids			Number of contigs with the indicated number of different insertion sites	
	
Grids	Insertions (N)	Percentage of total	Sites per contig	Contigs (N)
1	572	90%	1	48
2	60	9%	2	71
3	6	1%	3	89
4	1	0%	4	32
			5	7
			6	2
			7	1
Total	639	100%	Total	250

**Table 5:** Matching of *RescueMu *genomic loci to other available databases to determine percentage of genic and repeat loci

Category	Number of genomic loci
Total maize genomic loci discovered	14,265*
Number of genic loci identified by:	
Maize EST/cDNA	7,555
Plant EST/cDNA	1,253
Protein database	84 (62%)
GENSCAN prediction	708
Number of genic loci^† ^(percentage of total)	9,600 (67.3%)
	
Number of loci matching repeats:	
Retrotransposon	1,074
DNA transposon	212
MITEs	193
Centromere-related repeats	57
Telomere-related repeats	3
Unknown repeats	221
Other repeats	8
45S ribosomal DNA (18S + 28S)	23
5S ribosomal DNA	10
Transfer DNA	25
Number of repeat loci^‡ ^(percentage of total)	1,113 (8%)

*The 14,887 unique loci were collapsed into 14,265 unique loci by linking forward/reverse sequence pairs. This provides a more conservative estimate, but in some cases may have incorrectly combined separate loci. ^†^Numbers are cumulative: that is, GSSs were first matched to maize EST/cDNAs, then the unmatched GSSs were screened against other plant EST/cDNAs, and so on. ^‡^Numbers are not cumulative: that is, some loci could match to both retrotransposon and DNA transposon sequences.

**Table 6:** Single and multiple recovery of specific *RescueMu *insertion sites within the sequenced rows of grids G, H, I, K, M, and P

Grid	Single recovery	Multiple recoveries								
		
		0*	1^†^	2	3	4	5-9	10-19	20+	Total
G	3,801	22	640	326	62	20	17	2	2	4,892
H	2,142	13	331	136	31	9	6	3	6	2,677
I	2,000	10	348	124	39	3	9	6	6	2,544
K	1,000	6	155	50	11	2	1	2	1	1,228
M	1,075	3	225	74	13	5	5	3	2	1,405
P	1,731	8	246	100	27	5	22	1	1	2,141
All	11,749	62	1,945	810	183	44	60	17	17	14,887

Counts are of contigs containing sequences from the indicated number of rows. *The zero class represents plasmids identified in column sequencing that were not identified in any row. ^† ^The 1 column data include singlet plasmids as well as plasmids recovered two or more times but within a single row.

**Table 7:** Insertions found in at least two rows or columns among plants with both row and column sequence data

Grid	Plants from sequenced rows + columns	Insertions found in 2+ rows or 2+ columns
G	184	65
H	144	35
I	190	35
K	90	6
M	120	6
P	148	23
Total	876	177
