# Supplementary material for: Genome-wide mutagenesis of Zea mays L. using RescueMu transposons
Source: Genome Biol. 2004 Sep 23;5(10):R82. doi: 10.1186/gb-2004-5-10-r82 (PMC545602; doi:10.1186/gb-2004-5-10-r82)
Supplement: Additional data file 2 — Supplementary material for this paper, including details of methods [file gb-2004-5-10-r82-s2.doc]

Supplementary Materials for Genome-wide Mutagenesis of *Zea mays* L. using *RescueMu* Transposons

John Fernandes*, Qunfeng Dong^†^, Bret Schneider*^1^, Darren J. Morrow*, Guo-Ling Nan*, Volker Brendel^†^, and Virginia Walbot*^2^

*Department of Biological Sciences, Stanford University, Stanford, California 94305, ^†^Department of Zoology and Genetics, Iowa State University, Ames, IA 50011

^1^ Current address: United States Air Force, 55 LeMay Plaza South, Maxwell AFB, AL 36112-6335.

^2^ To whom correspondence should be addressed. E-mail: [Walbot@stanford.edu](mailto:Walbot@stanford.edu); fax 650-725-8221.

1. *RescueMu* Gene Tagging

**a. *RescueMu* Transgene Arrays and Somatic Excision Activity:** . *RescueMu* is a 1.4 kb *Mu1* element engineered to contain a pBluescript plasmid and a 400 bp segment from *Sinorhizobium meliloti* (previously named *Rhizobium meliloti*) (Raizada *et al.* 2001). The 4.7 kb *RescueMu* construct was inserted into the 5' untranslated region to block expression of a *35S:Lc* reporter allele. *Lc* is a member of the R family of transcription factors required for anthocyanin pigmentation. Using co-transformation with a herbicide resistance plasmid, multiple transgenic maize lines were recovered; in all lines, there were complex transgene arrays containing the *RescueMu* construct and the basta-resistance selection gene. By DNA blot hybridization, these arrays contained three to eight copies of the *35S:Lc::RescueMu* construct; each line yielded a characteristic and heritable restriction fragment profile (Raizada *et al.* 2001). Upon introduction of a transcriptionally active *MuDR* element(s), somatic excision of activated *RescueMu* from *35S:Lc* yielded mainly single cell sectors of red aleurone on a white *r-g* background. DNA sequence analysis indicated that short deletions and sequence insertions followed DNA repair after somatic excision (Raizada *et al.* 2001); these "footprints" are characteristic of somatic excision of *Mu1* at other mutable reporter alleles (Britt and Walbot 1991; Doseff *et al*. 1991). Therefore the somatic excision behavior of *RescueMu* in a complex transgene array parallels what is observed at host genes.

**b. Transgenic *RescueMu* Stocks -- mobilizing the element from transgene arrays:** Herbicide-resistant plants expressing the basta-resistance selection marker were confirmed by outcrossing to contain a closely linked *RescueMu* transgene array. These lines were crossed for two generations with *r-g* testers (A188 or W23 inbred lines or a W23/K55 hybrid). After crossing with an *r-g* active Mutator line, *RescueMu* excision from the 5' untranslated leader of a *35S:Lc* transgene could be scored as red aleurone sectors (Raizada *et al*. 2001). Additionally, Mutator lines with no evidence of *Mu* terminal inverted repeat (TIR) methylation and carrying other reporter genes were crossed to the *RescueMu* lines. Active Mutator lines with either a single copy of *MuDR* and the *a1-mum2* reporter allele (Robertson and Stinard 1989) or lines with a high number (>10) of *MuDR* elements and either the *bz1-mu1* (Britt and Walbot 1991) or *bz2-mu1* (McLaughlin and Walbot 1987) reporter alleles were used to activate *RescueMu* excision. These activated *RescueMu* lines were subsequently crossed to the appropriate anthocyanin tester lines to score somatic excision sectors from the standard reporter alleles.

As shown in Figure 1, the transgene arrays were inherited as expected (1:1 in a test cross with a standard anthocyanin tester line). Each array had a characteristic restriction digestion profile. Occasionally, a single plant contained a cross-hybridizing fragment smaller than 4.0 kb; subsequent analysis of several of these demonstrated that they were internally deleted *RescueMu* elements (lane 3, Figure 1).

____________________________________________________________________

**Figure 1**. DNA blot hybridization showing the segregation of the *RescueMu* transgene array and the presence of a shorter than expected element. Progeny of a plant heterozygous for the R3-8 transgene array were analyzed after *Hind*III digestion in which a 4.0 kb fragment is expected from the original *RescueMu* plasmid construct; the *Sinorhizobium* insert in *RescueMu* was used as the probe as it does not cross-hybridize with maize DNA. In the 17 progeny analyzed, 8 inherited the transgene array. One novel hybridizing fragment is smaller than 4.0 kb and likely represents a deleted element (yellow arrowhead). Fragments larger than 4.0 kb that were present in the original transgenic founder presumably represent rearranged elements within a complex array. Lane 1 has a novel ~12 kb fragment that could represent a deletion that eliminated a HindIII site within one copy of a *RescueMu* element (red arrowhead).


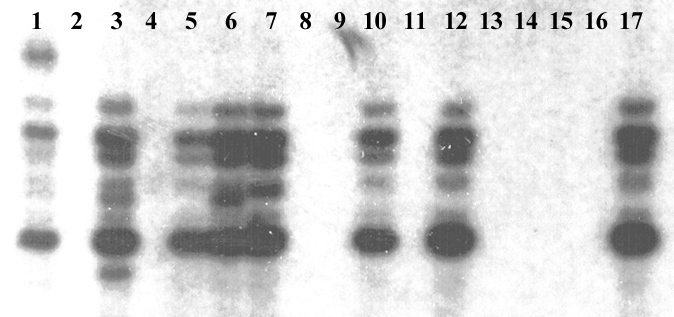


# - 4 Kb

_________________________________________________________________

*MuDR/Mu* elements cause a high frequency of germinal insertions late in the plant somatic life cycle, during meiosis, and in the gametophytes (Robertson 1981, 1989). Consequently, when spotted kernels indicative of somatic Mutator activity were selected and grown to maturity, we expected to find diverse *RescueMu* germinal insertions in the progeny. A total of 66 lines containing 8 independent transgene array loci were tested for overall *Mu*-induced forward mutation frequency and the frequency of *RescueMu* transposition in prototype Grid A (Table 1). *RescueMu* had been activated with diverse high copy Mutator lines and one type of single element line (Robertson and Stinard 1989). Lines were selected for further analysis only when they appeared to be fully active; for example, no evidence of *MuDR/Mu* element methylation and on crossing to the appropriate anthocyanin testers, somatic excision was observed at the *a1-mum2*, *bz2-mu1*, or *bz1-mu1* reporter alleles, as appropriate (data not shown). DNA blot analysis of multiple siblings to each line in Grid A identified far fewer putative *trRescueMu* than expected. Most families examined had no *trRescueMu*. In the survey blot shown in Figure 1, plant 3 has a novel band, but this is a deleted form of *RescueMu*. In a total of 527 seedlings examined, only 39 *trRescueMu* were identified, and all of these were from lines with multiple copies of *MuDR*.

_____________________________________________________________________

**Table 1.** Characteristics of lines represented in prototype Grid A. Grid A was grown in Hawaii in winter 1999 to evaluate the efficacy of tagging from eight different transgene loci; in some cases, four of the transgene loci were tested in two Mutator backgrounds, one with a single copy of *MuDR* and one with multiple copies.

| Transgene locus | Number of families | *MuDR* copy number | Individuals screened | Percent siblings with *trRescueMu*^a^ |
| --- | --- | --- | --- | --- |
| R3-3 | 1 | 1 | 10 | 0 |
| R3-4 | 3 | 1 | 16 | 0 |
| R3-4 | 16 | ~10 | 51 | 5 |
| R3-7 | 1 | 1 | 20 | 0 |
| R3-8 | 3 | 1 | 20 | 0 |
| R3-8 | 18 | ~10 | 108 | 11 |
| R3-12 | 1 | ~10 | 20 | 0 |
| R3-13 | 2 | 1 | 20 | 0 |
| R3-13 | 11 | ~10 | 80 | 15 |
| R3-15 | 2 | 1 | 20 | 0 |
| R3-15 | 1 | ~10 | 12 | 0 |
| R3-17 | 7 | ~10 | 150 | 8 |
| Total | 66 |  | 527 | 39 |

^a^ Individuals of each family were tested by DNA blot hybridization for the presence of *trRescueMu*. Fragments smaller than the length of an intact *RescueMu* were scored as deletions and not counted as transposed copies. In a few cases such smaller elements were recovered by plasmid rescue and demonstrated to contain deletions (data not shown).

____________________________________________________________________

Putative full-length *trRescueMu* were recovered by plasmid rescue and subsequent sequencing of the flanking host DNA demonstrated the presence of a 9 bp host sequence duplication characteristic of *Mu* insertion events as well as new flanking host DNA (Raizada *et al.* 2001). Considering just the four families with at least one transposition event, there is a 10% frequency of *trRescueMu* (39/389). Therefore, most Grid A plants are unlikely to contain a *trRescueMu*. In contrast to the low frequency of *trRescueMu*, many new *Mu1* insertion sites could be visualized, as expected from the robust mutation frequency observed in the selfed progeny of most Grid A families (discussed in the next paragraph). Thus, the *Mu* elements were actively transposing but the *RescueMu* elements were transposing at a much lower frequency. Furthermore, digestion of genomic DNA with the methylation sensitive enzyme *Sac*I produced the expected 4.7 kb *MuDR* fragment (data not shown) indicating that epigenetic silencing had not occurred.

Among the 1536 progeny ears obtained by selfing individuals in Grid A, 7.2% of the ears had a new, segregating seed mutation, 4.5% of families exhibited a new seedling mutation, and 7.9% of the progeny displayed a new adult mutation (see Table 1 in the manuscript for details of all grids). The 19 families in Grid A with only a single copy of *MuDR* contributed few new mutations, and they did not program germinal transposition of *RescueMu*. Subtracting these single copy *MuDR* families, the frequency of mutations in the multi-copy *MuDR* lines was similar to Robertson's 10% seedling mutation frequency criterion for a highly active Mutator line (Robertson 1985).

**c. Testing the Best Transgene Array Lines as Source Materials for Large Scale Tagging:** *RescueMu* excises somatically at high frequency from the *35S:Lc* construct, demonstrating that the *Mu* termini are accessible to MURA transposase in somatic cells. It was consequently surprising and disappointing that the germinal transposition frequency was so low in Grid A. To determine if there were line-specific factors that impeded germinal transposition, individuals related to those from the best-performing lines in Grid A were grown as Grids B through E in 1999 at four locations. These grids contained only high copy *MuDR/Mu* lines, and the *RescueMu* transgene arrays had been activated for a minimum of two generations. As shown in Table 1 in the accompanying paper, these four grids yielded higher forward mutation frequencies; indeed, Grids C and E were nearly three times more active in generating new, independent seedling mutations than previously well-characterized Mutator lines (Walbot and Rudenko 2002). Despite the high forward mutation frequency in seed and seedling visible traits, an accompanying survey of 30 to 200 plants within each of these grids verified only a low frequency of new *trRescueMu* elements (Table 2). Epigenetic loss of Mutator activity was ruled out as the explanation for the sluggish transposition of *RescueMu* based on several criteria. First, somatic excision from reporter genes remained high. Second, few individuals examined showed methylation in *Mu* TIRs (data not shown). Third, standard *MuDR/Mu* elements were active in germinal transposition measured as forward mutation frequency (Table 1, manuscript).

**d. Use of Plants with *trRescueMu* as Founders for High Frequency Transposon Tagging:** Given the low efficiency of *RescueMu* mobilization from 8 different transgene arrays combined with diverse, highly active Mutator lines, a new strategy was designed. Progeny of plants with a single *trRescueMu* were screened to identify individuals with several new *trRescueMu* elements, indicative of increased copy number. Lines that contained a *trRescueMu* but had lost the transgene array, as indicated by herbicide sensitivity, were of particular interest. The herbicide-sensitive individuals and their progeny were screened by DNA blot hybridization to identify and eliminate lines that contained *trRescueMu* elements shorter than full-length. These surveys also verified that individual *trRescueMu* were germinal (heritable) insertions and not cases of somatic insertion in a leaf sector used for the DNA analysis in the previous generation. Verified full-length, heritable *trRescueMu* were designated as parental elements in a subsequent cross.

To test the ability of parental *trRescueMu* to transpose again in an active Mutator line, a population of 393 plants representing descendants of four different cases of *trRescueMu* was screened by DNA blot hybridization (Table 2).

___________________________________________________________________

**Table 2. DNA blot hybridization survey for newly *trRescueMu* in lines with a single parental transposed copy and no transgene array.**

| Family | Individuals | Progeny with *trRescueMu*  Number of new elements^a^  1 2 3 | | | Insertion per  Plant (%) |
| --- | --- | --- | --- | --- | --- |
| M805 | 23 | 5 | 2 |  | 39 |
| M815 | 22 | 1 |  | 1 | 18 |
| M816 | 20 |  |  |  | 0 |
| M817 | 15 | 2 | 2 |  | 27 |
| M818 | 14 |  |  |  | 0 |
| M819 | 17 |  |  |  | 0 |
| M820 | 16 | 3 |  |  | 19 |
| M821 | 12 | 1 | 1 | 1 | 50 |
| M822 | 20 |  |  |  | 0 |
| M823 | 22 | 1 |  |  | 4 |
| M825 | 13 |  |  |  | 0 |
| M826A,B | 8 | 1 |  |  | 13 |
| M828A | 2 | 2 |  |  | 100 |
| M828B | 22 |  |  |  | 0 |
| M829A | 11 |  | 1 | 1 | 45 |
| M829B | 11 | 1 | 2 |  | 45 |
| M829C | 15 | 1 |  |  | 7 |
| M829D | 22 | 3 | 1 | 1 | 36 |
| M830 | 14 |  |  |  | 0 |
| M831 | 3 | 1 |  |  | 33 |
| M832B | 21 |  |  |  | 0 |
| M833 | 20 | 1 |  | 1 | 20 |
| M834 | 13 | 1 |  | 2 | 61 |
| M835A | 9 | 3 |  |  | 33 |
| M835B | 10 | 2 | 1 |  | 30 |
| M835C | 18 | 1 | 1 |  | 11 |
| Total | 393 | 30 | 11 | 7 | 18.6 |

^a^DNA samples were singly or doubly digested with restriction enzymes with one recognition site in *RescueMu.*  Novel fragments larger than the minimum size of *RescueMu* were counted as transposition events. Given the late timing of most *Mu* germinal insertions (Robertson 1981, 1989), individual cases of newly transposed elements were expected to be present in only one or a few plants within a family, and this was observed in the DNA blot surveys (data not shown). As shown in Figure 2 in the accompanying paper, parental *trRescueMu* elements segregate in a family and numerous individuals contain a new *trRescueMu.*

____________________________________________________________________

Altogether 7 individuals were identified that contained 3 new putative *trRescueMu*, and 7 individuals were identified with 2 putative germinal insertion events. An additional 23 individuals contained a single, non-parental *trRescueMu* (Table 2). Thus, the overall germinal insertion frequency was 18.6% (73/393) on a per plant basis. If the families with no *trRescueMu* are excluded from the analysis the germinal insertion frequency was 26% (73/280) with a range of 7 to 100% transpositions per element. For a subset of families lacking *trRescueMu* in this experiment, DNA blot hybridization was used to check the methylation status of *Mu1* and/or *MuDR* elements. Epigenetic loss of Mutator activity, as reflected in methylation within the TIRs, does not explain the low mobility of *trRescueMu* in the families tested (data not shown). We have identified no explanation for the significant differences in transposition frequency between lines containing individual *trRescueMu* elements*.*

Founder individuals with two or three *trRescueMu* elements were crossed multiple times as pollen parent to recipient non-Mutator lines to produce seed for grids F through J. The progeny ears were grouped by date of pollination, 4 to 20 kernels were selected per pollination date, and DNA was prepared from individual germinating seeds. By DNA blot hybridization after HindIII or other restriction enzyme digestion, the segregation of the parental *trRescueMu* elements from the founder male was scored along with the presence of newly *trRescueMu* elements. In some cases a second restriction digestion using either Hinf1 or Sst1 was conducted to determine the methylation status of *Mu1* plus *Mu2* or of *MuDR* elements, respectively. To constitute a grid, kernels were selected from the population of ears from a single founder individual with the highest transposition of *RescueMu* and no evidence of Mutator silencing. From 50 to 200 individual plants within Grids F through J were screened for new *trRescueMu* to obtain a better estimate of transposition frequency. After DNA blot hybridization to verify the absence of *MuDR/Mu* methylation, plants with two or several new *trRescueMu* were used as individual male founder plants to generate the tagging populations used in Grids K through O. Similarly, founder individuals were identified among individuals in these grids to build subsequent grids. Grid P was based on a single pollen donor with 3 parental *trRescueMu*, and Grids Q and R were based on a single pollen donor with 2 *trRescueMu*. Grids S, T, and U contained progeny from multiple, related founder plants with two or three *trRescueMu*. Grids AA and BB were constructed by sib-crossing related plants with several *trRescueMu* in an effort to obtain tagged alleles of genes that could confer pollen abortion.

**e. Tagging Grids:** Populations of up to 3000 seed were planted, and juvenile or young adult plants were chosen for grids of up to 2304 plants. These were organized into 48 rows and 48 columns, and each plant was assigned a unique row and column address. Losses to weather, vandalism, and/or animals reduced the grid size in many cases. The intent was to self-pollinate each grid plant to permit scoring of segregating recessive traits in the progeny; in cases of male or female sterility, plants were outcrossed to anthocyanin tester lines or sib-crossed to extra plants available from the grid planting. Harvested ears were shipped to the Maize Genetics Cooperation – Stock Center (Maize COOP; <http://www.uiuc.edu/ph/www/maize)>. Stocks with <30 seed are expanded by the Maize COOP staff prior to distribution; outcrossed stocks are sib-crossed to generate individuals homozygous for *Mu* insertions.

**f. DNA blot hybridization protocols:** Segments of about 2 by 6 cm were removed from juvenile or adult leaf blades, kept ice-cold if used immediately or stored at -80^o^C, and then pulverized in liquid nitrogen by grinding in a mortar and pestle or with a glass rod inserted in a 50 mL plastic tube on a vortex mixer. Genomic DNA was extracted by a modified urea method (Chen and Dellaporta 1994) or a CTAB method (Dellaporta 1994). Using the restriction digestion protocols described in Raizada *et al*. (2001), samples were scored for the presence of *RescueMu* after gel electrophoresis and DNA blot preparation using standard methods. Blots were probed with either a *S. meliloti* fragment or a fragment of the pBluescript ampicillin resistance gene, using standard procedures. Probes were labeled with ^32^P by nick-translation (Sambrook *et al.* 1989) or with the AlkPhos DIRECT^TM^ labeling and chemiluminescence detection system (Amersham Biosciences, Inc., Piscataway, NJ, USA). To identify *trRescueMu*, several different restriction enzyme digestion strategies were employed. Most commonly, either *EcoR*I or *Hind*III were used, because each has a unique recognition site near one end of *RescueMu* and new insertion sites should yield fragments larger than 4.0 kb. Shorter than expected *RescueMu* hybridizing fragments were often found and were scored as deleted *trRescueMu* (Figure 1). *Hin*fI digestion was used to score methylation status in the terminal inverted repeats (TIRs) of the closely related *Mu1* and *Mu2* elements using internal segments of *Mu1* for detection (Chandler and Walbot 1986). Digestion with *Sst*I was used to score the methylation status of *MuDR* elements, which contain this methylation-sensitive restriction site in each TIR (Martienssen *et al*. 1990), using internal segments of *MuDR* as the probe. For DNA blot hybridization surveys for deletions within *MuDR*, the methylation-insensitive isoschizomer SacI was used in some cases. For lines with no discernible methylation in the TIRs, the intensity of hybridization signal of the expected size (1.3 for *Mu1*, 1.6 for *Mu2*, and 4.7 for *MuDR*) was used to estimate the copy number of *Mu* elements; this was done by comparison to plasmid reconstruction standards (Walbot and Warren 1988) or by comparison to a line with a single genetically active copy of *MuDR* and *Mu1* (Robertson and Stinard 1989; Chomet *et al*. 1991).

**2. Plasmid Rescue, DNA Sequencing Protocols, and Insertion Site Analysis**

**a. Plasmid Rescue:** Briefly, total maize DNA was extracted, digested with *Bgl*II and *BamH*I, incubated with DNA ligase, and then used to transform recipient *Escherichia coli* by electroporation. Only *E. coli* receiving a maize DNA segment containing *RescueMu* should survive during ampicillin selection. A detailed protocol is presented at [http://www.mutransposon.org/project/RescueMu/zmdb/protocols/rmu/plasmid_rescue.html](http://www.zmdb.iastate.edu/zmdb/protocols/rmu/plasmid_rescue.html)

**b. DNA Sequencing of *RescueMu* Plasmid Genomic Inserts:** Detailed protocols are available for colony picking at <http://www.mutransposon.org/project/RescueMu/zmdb/protocols/rmu/picking.html> and the PCR amplification of templates at [http://www.mutransposon.org/project/RescueMu/zmdb/protocols/rmu/pcr.html](http://www.zmdb.iastate.edu/zmdb/protocols/rmu/pcr.html)

These templates were subjected to cycle sequencing as described at [http://www.mutransposon.org/project/RescueMu/zmdb/protocols/rmu/cycle_sequencing.html](http://www.zmdb.iastate.edu/zmdb/protocols/rmu/cycle_sequencing.html)

and then loaded onto MegaBACE1000 96-well capillary electrophoresis DNA sequencing machines as described at [http://www.mutransposon.org/project/RescueMu/zmdb/protocols/rmu/loading_dna_samples.html](http://www.zmdb.iastate.edu/zmdb/protocols/rmu/loading_dna_samples.html)

Interpretation of sequencing results -- particularly the use of the 9 bp host sequence duplication to join the host sequence information from the right and left sides of *RescueMu* and the method of reporting and assembling sequences after encountering a *BamH*I, *Bgl*II, or ligation motif generated by these enzymes -- is presented at <http://www.zmdb.iastate.edu/zmdb/library-plate/GridGprep.html>

c. *RescueMu* Element Sequencing

Although *RescueMu* was assembled from the individual segments of defined sequence, the final construct was not sequenced, although a detailed restriction map was checked (Raizada *et al.* 2001). To verify the precise sequence of the 4.7 kb element, 15 pairs of PCR primers were designed to amplify overlapping segments for sequencing. Bidirectional sequencing was conducted to generate a minimum of 7 sequence reads for every base. The consensus sequence is reported in GenBank (Accession AY301066).

______________________________________________________________________

Table 3. Primers used for PCR amplification across *RescueMu*

*RescueMu* is a modified *Mu1* element, and the base numbering utilizes the standard orientation of this element with the left TIR containing base #1 on the upper strand. Primers on the lower strand are listed with the corresponding base numbers from the upper strand.

| Primer  Set | Starting Base | Ending Base | Upper strand sequence, 5' to 3' | Note |
| --- | --- | --- | --- | --- |
| L1 | 219 | 242 | CGCTCTACGGCAGCACGGCGGTGCT | Read out through TIR |
| L2 | 28 | 48 | GGAAGGGGATTCGACGAAATG | Within TIR |
| L3 | 393 | 408 | GGACCTCCTCCTCCCG | Through rhizobium sequence |
| L4 | 867 | 887 | GCCACTCCTATATAGCCTCAC | Rhizobium to pBluescript |
| L5 | 1048 | 1065 | CAACGTCATCTCGGCTTC | pBluescript |
| L6 | 1414 | 1435 | CAACAGTTGCGCAGCCTGAATG | pBluescript |
| L7 | 1777 | 1794 | ACGTGGCGAGAAAGGAAG | pBluescript |
| R8 | 2818 | 2836 | GGAGTCAGGCAACTATGGA | pBluescript |
| R7 | 3272 | 3291 | CGCTCTGCTAATCCTGTTAC | pBluescript |
| R6 | 3453 | 3470 | CTATGAGAAAGCGCCACG | pBluescript |
| R5 | 3593 | 3610 | CCTCTGACTTGAGCGTCG | pBluescript |
| R4 | 4161 | 4179 | GGCTCTTCCAGCAGAGTAG | pBluescript |
| R3 | 4518 | 4534 | GACTGAGATGCGACGGA | Mu1 |
| R2 | 4692 | 4712 | TATTTCGTCGAATCCGCTTCT | Mu1 |
| R1 | 4513 | 4535 | CGCGTGACTGAGATGCGACGGAG | Out through TIR |

**d. PCR Screening of Library Plates:** Library plates contain the row and column *E. coli* plasmid rescue libraries prepared from an individual grid. A detailed protocol for library plate construction is presented at

http://www.mutransposon.org/project/RescueMu/zmdb/protocols/rmu/library_plate_production.html

An overview of the use of library plates is described at http://www.zmdb.iastate.edu/zmdb/library-plate/

Screening library plates by PCR to identify row and column matches is described at <http://www.zmdb.iastate.edu/zmdb/library-plate/screening.html>

Because different leaves were sampled for the row and column tissue collections, somatic insertions are expected in nearly all cases to be found in a row or a column library only. Germinal insertions, which are inherited from the founder plant, should be present in both the row and column library for a particular plant. Approximately 20 - 40% of *RescueMu* insertions appear to occur as small somatic sectors in the tassel of a founder plant; these insertions will be present in more than one row or more than one column, or in rare cases of a very early somatic sector in multiple rows and multiple columns. *RescueMu* plasmids were sequenced from DNA libraries prepared from grid rows and 4 columns. If the same *RescueMu* insertion site is found from a row library and from a column library of the same grid (as noted from a blast search at GenBank or in a RescueMu sequence assembly at ZmDB), the data specify which plant contains that insertion, i.e. Grid G row 12 column 15. Furthermore, representation in both a row and a column library is strong evidence of a germinal insertion.

For most *RescueMu* insertion locations, only row sequence data area available. Using information on sequenced *RescueMu* plasmids from a particular grid, PCR primers from a gene of interest can be designed within 200 - 500 bp of a particular *RescueMu* insertion site. If the known *RescueMu* insertion was reported in row 7, for example, then screening the library plate of up to 96 libraries should confirm that row 7 is positive. For a germinal insertion, a column(s) should also be positive. Both the row and column products should be of the same size and the size expected from the known sequence.

Because grid sequencing was always incomplete, many germinal insertions are represented by a single *RescueMu* plasmid sequence. There is increasing confidence that a germinal insertion exists if two or more sequences of the same plasmid were obtained.

Each library plate contains ~10,000 to ~20,000 different *RescueMu* plasmids, the sum of somatic, germinal and parental insertion sites. The average insert size in Grid G was 3.4 kb, but the range was 0.6 to 15 kb. Surveys of ~96 plasmids from subsequently sequenced grids indicated a similar range. Consequently, sequencing next to *RescueMu* insertion sites rarely provided the entire genomic sequence in a particular plasmid. Plasmids recovered from library plates, by plating and screening the collection, will in many cases provide templates for sequencing of an entire gene and its flanking regulatory regions.

**e. Ordering Seed from the Maize Coop:** <http://www.uiuc.edu/ph/www/maize>

Seed are freely available from the Maize Coop. Because *RescueMu* is a transgene, seed requests must be accompanied by an APHIS certification that shipping has been approved from the state of Illinois to the requestor in another state. For requests from outside the USA, the requester must inquire about any regulatory requirements from their government, and inform the Maize COOP staff of the proper procedures.

**f. Verifying Insertion Mutations in Seed:** PCR or DNA blot hybridization survey should be conducted on plants derived from the selfed seed sample to identify those individuals homozygous for the *RescueMu* insertion of interest and to identify heterozygous and "wild type" siblings. After inspection of plant phenotypes, association of segregating visible mutation with homozygosity for the *RescueMu* insertion can be determined. If no homozygous individuals are identified it is likely that the *RescueMu* or a linked *Mu* insertion causes male or female sterility.

**3. Phenotypic Analysis of Selfed Progeny of *RescueMu* Tagging Grids**

**a. PhenotypeDB:** <http://www.zmdb.iastate.edu/zmdb/phenotypeDB/index.htm>

This searchable database contains descriptions of all visible mutations identified by the Maize Gene Discovery Project in evaluating the selfed progeny of grid plants. The database contains photographs of novel mutations and is linked to the DNA sequence data from *RescueMu* plasmids of the corresponding sequenced rows or columns.

______________________________________________________________________

**Table 4.** Progress in phenotypic screening of *RescueMu* tagging grid progeny.

| Stage | Completed  Spring 2003 | Future Plans |
| --- | --- | --- |
| Ear & seed | 35,000 | Screen complete set of ears from all  Grids (approx. 45,000 ears) |
| Seedling | 9,000 | ~3,000 families per year drawn mainly from sequenced grids |
| Adult | 7,000 | ~3,000 families in 2004 and 2005 |

______________________________________________________________________

The *RescueMu* tagging grid populations were carefully selected for a high transposition frequency of *RescueMu*, and this was accompanied by a high rate of transposition of standard *Mu* elements. The tagging populations have the highest forward mutation frequencies yet reported for Mutator lines. Although only a subset of visible mutations are caused by *RescueMu*, nearly all of the mutations scored are expected to result from *Mu* insertions. To capture a robust description of the phenotypes segregating after self-pollination of grid individuals, the resulting progenies were screened at three stages of development: mature ear and kernels, germinating seedling through ~5 weeks of growth in a greenhouse sand bench, and the full life cycle in a field setting.

**b. Scoring Mutation Frequency:** Because of the genetic lineages that went into making up a grid, it was commonplace for particular mutations -- occurring in prior generations in the *Mu*-active lineage --to be represented in more than one family. These parental or grandparental mutations were noted, but calculation of forward mutation frequency was based on recording a segregating phenotype as a “new recessive mutant” once. This frequency varied from 6.9% (Grid H) to 28% (Grid C). Mutant phenotypes occurring once in a family were designated “possibly dominant.” This number was approximately 25% of the new mutant frequency. We suspect that the majority of “possibly dominant” mutants are actually recessive mutants with low penetrance, or combinations of recessive mutants occurring in our varied backgrounds. This hypothesis is being tested.

**c. Scoring Ear and Kernel Traits:** Seed set, kernel color, and other phenotype descriptions are recorded for each ear in every grid. Seed set varied from normal (complete kernel fill and in complete straight rows) to a semi-sterile appearance (scattered kernel placement lacking full rows). Kernel and pericarp colored or colorless status were recorded, then a closer examination was done on individual kernels to search for any subtle color variations or patterns. The search for mutant phenotypes included observation of kernel size and shape, particularly on the crown, noting kernels noticeably different than the normal majority of kernels on that ear. If an abnormal kernel class existed, two or more variant kernels were removed for closer examination to score the presence of an embryo and to examine endosperm development. For kernels with an aberrant endosperm, an iodine stain was used to determine starch quality. Each ear was also examined for cob (maternal) mutations, including defects in the glumes.

**d. Greenhouse Observation of Seedling Traits:** Sixteen seeds per F2 family were grown in flats on the surface of 8 cm of vermiculite, held down by about 1 cm of sand. Greenhouses rarely fell below 23 °C, but daytime temperatures fluctuated, as there was no cooling beyond ventilation. Light was extended by metal-halide illumination until 10 pm each evening. Flats were watered to saturation three times per day, with nutrients added during once per day in the form of 1/2-strength Hoaglands augmented with 2X calcium, in the form Ca(NO_3_)_2_.4H_2_O, three times per week. Hot summer days required more water. Coleoptiles emerged within five days. Plants were screened periodically for approximately 5 to 6 weeks. Phenotypic data were noted, sometimes graphically. Some possibly dominant mutants were transplanted for propagation. Our textual data was abbreviated using combinations of words from an established glossary available at ZmDB: <http://www.zmdb.iastate.edu/zmdb/phenotypeDB/index.htm> In order to maintain consistency in the more subjective aspects of mutant screening, M. Freeling observed each seedling and proofed all data sent to PhenotypesDB.

**d. Field Observation of Plant Traits:** An initial assessment of ~1100 adult families was made at UC-San Diego using materials from Grid A by scoring ~20 individuals per family. The second adult plant observation plot consisted of 3,000 16-foot long rows, each planted with 25 sibling kernels from families from Grids C, E, and J. These materials were evaluated in summer 2001 at the University of Illinois. The plants were observed for traits approximately every 7 days by members of the Maize COOP staff; these observations started shortly after plant emergence and continued until just prior to tassel emergence. An intensive pre-flowering screen was conducted by Maize Gene Discovery project members and focused on all plant traits except those of the ear and tassel. Comparisons were within each individual family, noting individuals that differed from their siblings in plant color, height, organ morphology, or expression of novel traits. Traits such as leaf size, width, presence of additional or missing plant tissue, variations in leaf margins, or any variation in surface appearance or texture were noted. Differences in sheath size, or stalk rigidity, or stature were also noted*.* At flowering, ear placement, husk morphology, presence of silks, tassel structure, pollen abnormalities, and male sterility were scored. Deviations in ear morphology were noted as part of the ear and kernel screen. In summer 2002 a second screen of similar magnitude was conducted with materials from Grids F, G, K, and L, and screens are planned for summers 2003 (3308 families from Grids P, Q, and BB) and 2004.

4. Additional Information about the Bioinformatics Analysis

**a. Number of Loci for indicated number of rows/columns for all Grids.**

|  | | **Columns** | | | | | | |
| --- | --- | --- | --- | --- | --- | --- | --- | --- |
| Grid | **Rows** | | **0** | **1** | **2** | **3** | **4** | **5** |
| G | 0 | |  | 282 | 1 |  |  |  |
| G | 1 | | 4097 | 82 | 1 |  |  |  |
| G | 2 | | 291 | 33 | 2 |  |  |  |
| G | 3 | | 47 | 14 | 1 |  |  |  |
| G | 4 | | 15 | 5 |  |  |  |  |
| G | 5-9 | | 12 | 3 | 2 |  |  |  |
| G | 10-19 | |  | 1 |  | 1 |  |  |
| G | 20+ | |  |  | 1 |  | 1 |  |
| G | All | |  |  |  |  |  |  |
| H | 0 | |  | 211 |  |  |  |  |
| H | 1 | | 2225 | 49 | 1 |  |  |  |
| H | 2 | | 122 | 13 | 1 |  |  |  |
| H | 3 | | 26 | 5 |  |  |  |  |
| H | 4 | | 4 | 5 |  |  |  |  |
| H | 5-9 | | 3 | 2 |  | 1 |  |  |
| H | 10-19 | |  | 2 |  | 1 |  |  |
| H | 20+ | | 1 |  | 1 | 1 | 2 |  |
| H | All | |  |  |  |  | 1 |  |
| I | 0 | |  | 230 | 1 |  |  |  |
| I | 1 | | 2066 | 61 |  |  |  |  |
| I | 2 | | 108 | 15 | 1 |  |  |  |
| I | 3 | | 31 | 8 |  |  |  |  |
| I | 4 | | 1 | 1 | 1 |  |  |  |
| I | 5-9 | | 7 | 2 |  |  |  |  |
| I | 10-19 | | 3 | 2 |  |  | 1 |  |
| I | 20+ | |  |  |  |  | 2 | 2 |
| I | All | |  |  |  |  |  | 1 |
| K | 0 | |  | 84 |  |  |  |  |
| K | 1 | | 1056 | 21 |  |  |  |  |
| K | 2 | | 47 | 3 |  |  |  |  |
| K | 3 | | 9 | 2 |  |  |  |  |
| K | 4 | | 1 | 1 |  |  |  |  |
| K | 5-9 | | 1 |  |  |  |  |  |
| K | 10-19 | | 2 |  |  |  |  |  |
| K | 20+ | |  |  |  |  |  |  |
| K | All | |  |  |  | 1 |  |  |
| M | 0 | |  | 60 | 1 |  |  |  |
| M | 1 | | 1223 | 19 |  |  |  |  |
| M | 2 | | 71 | 3 |  |  |  |  |
| M | 3 | | 11 | 2 |  |  |  |  |
| M | 4 | | 5 |  |  |  |  |  |
| M | 5-9 | | 4 |  |  | 1 |  |  |
| M | 10-19 | | 3 |  |  |  |  |  |
| M | 20+ | | 1 |  |  |  |  |  |
| M | All | |  |  |  | 1 |  |  |
| P | 0 | |  | 135 |  |  |  |  |
| P | 1 | | 1828 | 22 |  |  |  |  |
| P | 2 | | 92 | 8 |  |  |  |  |
| P | 3 | | 20 | 7 |  |  |  |  |
| P | 4 | | 5 |  |  |  |  |  |
| P | 5-9 | | 16 | 4 |  |  | 2 |  |
| P | 10-19 | |  |  | 1 |  |  |  |
| P | 20+ | |  |  | 1 |  |  |  |
| P | All | |  |  |  |  |  |  |
| Total | 14887 | | 13454 | 1397 | 17 | 7 | 9 | 3 |

5. Acknowledgments.

The Maize Gene Discovery Project was funded by the National Science Foundation. The laboratories of Marty Sachs, Mike Freeling, Bob Schmidt, and Sarah Hake performed the phenotypic screening. Laura Roy designed and edited PhenotypeDB. Molecular analysis of trRescueMu was performed in the laboratories of Vicki Chandler, Sarah Hake, Bob Schmidt, and Virginia Walbot. Diane Chermak in the Walbot laboratory generated the *RescueMu* sequencing templates and library plates. Nausica Arnout and Katrina Goellner, visiting students in the Walbot lab, utilized the PCR library plate screening protocol to verify germinal transmission of sequenced *trRescueMu* insertion sites as described in the protocols.

6. References

Britt, A. B., and V. Walbot, 1991 Germinal and somatic products of excision of *Mu1* from the *Bronze-1* gene of *Zea mays*. Mol. Gen. Genetics **227:** 267-276.

Chandler, V. L., and V. Walbot, 1986 DNA modification of a maize transposable element correlates with loss of activity*. Proc. Natl. Acad. Sci. USA* **83**:1767-1771.

Chen, J. and S. Dellaporta 1994 Urea-based plant DNA miniprep, pp. 522-525 in *The Maize Handbook* edited by M. Freeling and V. Walbot. Springer-Verlag, New York.

Chomet, P., D. Lisch, K. J. Hardeman, V. L. Chandler, and M. Freeling, 1991 Identification of a regulatory transposon that controls the *Mutator* transposable element system in maize. Genetics **129:** 261-270.

Dellaporta, S., 1994 Plant DNA miniprep and microprep: Version 2.1-2.3, pp. 522-525 in *The Maize Handbook* edited by M. Freeling and V. Walbot. Springer-Verlag, New York.

Doseff, A., R. Martienssen, and V. Sundaresan, 1991 Somatic excision of the *Mu1* transposable element of maize. Nucl. Acids Res. **19:** 579-584.

Martienssen, R., A. Barkan, W. C. Taylor, and M. Freeling, 1990 Somatically heritable switches in the DNA modification of *Mu* transposable elements monitored with a suppressible mutant in maize. Genes Dev. **4:**331-343.

McLaughlin, M. and V. Walbot, 1987 Cloning of a mutable *bz2* allele of maize by transposon tagging and differential hybridization. Genetics **117:** 771-776.

Raizada, M. N., G. L. Nan and V. Walbot, 2001 Somatic and germinal mobility of the *RescueMu* transposon in transgenic maize. Plant Cell **13:** 1587-1608.

Robertson, D. S., 1981 Mutator activity in maize: Timing of its activation in ontogeny. Science **213:** 1515-1517.

Robertson, D. S. 1985. Differential activity of the maize mutator *Mu* at different loci and in different cell lineages. Mol. Gen. Genet. **200:** 9-13.

Robertson, D. S., 1986 Genetics studies on the loss of *Mu* Mutator activity in maize. Genetics **113:**  765-773.

Robertson, D. S., 1989 The timing of *Mu* activity in maize. Genetics **94:** 969-978.

Robertson 1989

Robertson, D. S. and P. S. Stinard, 1989 Genetic analyses of putative 2-element systems regulating somatic mutability in Muator-induced aleurone mutations of maize. Dev. Gen. **10:** 482-506.

Sambrook, J., E. F. Fritsch, and T. Maniatis, 1989 Molecular cloning: a laboratory manual, 2^nd^ Edition. Cold Spring Harbor Laboratory, Cold Spring Harbor NY.

Walbot, V. and G. N. Rudenko, 2002 *MuDR/Mu* transposons of maize. In: **Mobile DNA II**, eds. N. L. Craig, R, Craigie, M. Gellert, A. Lambowitz. Amer. Soc. Microbiology, Washington, D. C. pp. 533-564.
